# Sex difference in human diseases: mechanistic insights and clinical implications

**DOI:** 10.1038/s41392-024-01929-7

**Published:** 2024-09-10

**Authors:** Yuncong Shi, Jianshuai Ma, Sijin Li, Chao Liu, Yuning Liu, Jie Chen, Ningning Liu, Shiming Liu, Hui Huang

**Affiliations:** 1 Department of Cardiology, the Eighth Affiliated Hospital, Joint Laboratory of Guangdong-Hong Kong-Macao Universities for Nutritional Metabolism and Precise Prevention and Control of Major Chronic Diseases, Sun Yat-sen University, Shenzhen, China; 2 Department of Radiotherapy, Sun Yat-sen Memorial Hospital, Sun Yat-sen University, Guangzhou, China; 3 Department of Cardiology, Guangzhou Institute of Cardiovascular Disease, Guangdong Key Laboratory of Vascular Diseases, the Second Affiliated Hospital, Guangzhou Medical University, Guangzhou, China

**Keywords:** Cardiology, Cardiovascular diseases

## Abstract

Sex characteristics exhibit significant disparities in various human diseases, including prevalent cardiovascular diseases, cancers, metabolic disorders, autoimmune diseases, and neurodegenerative diseases. Risk profiles and pathological manifestations of these diseases exhibit notable variations between sexes. The underlying reasons for these sex disparities encompass multifactorial elements, such as physiology, genetics, and environment. Recent studies have shown that human body systems demonstrate sex-specific gene expression during critical developmental stages and gene editing processes. These genes, differentially expressed based on different sex, may be regulated by androgen or estrogen-responsive elements, thereby influencing the incidence and presentation of cardiovascular, oncological, metabolic, immune, and neurological diseases across sexes. However, despite the existence of sex differences in patients with human diseases, treatment guidelines predominantly rely on male data due to the underrepresentation of women in clinical trials. At present, there exists a substantial knowledge gap concerning sex-specific mechanisms and clinical treatments for diverse diseases. Therefore, this review aims to elucidate the advances of sex differences on human diseases by examining epidemiological factors, pathogenesis, and innovative progress of clinical treatments in accordance with the distinctive risk characteristics of each disease and provide a new theoretical and practical basis for further optimizing individualized treatment and improving patient prognosis.

## Introduction

Sex represents a significant physiological factor impacting human growth, development, and behavior, with implications across various bodily systems. Sex disparities leaded to differences in disease incidence, clinical features, physiological mechanisms, and response to treatment.^1^ For instance, women had a higher susceptibility to hypothyroidism and autoimmune diseases, while men faced an elevated risk and mortality rate of developing cancer.^2,3^ Growing evidence suggested that physiological and environmental factors participated in sex disparities within cardiovascular, neoplastic, metabolic, immune, and nervous systems. Sex hormones, sex chromosomes, and epigenetic factors regulated gene expression, receptors, and signaling pathways, thereby influencing sex-specific neurobiology and pathological manifestations.^4^ Over the years, heart failure has remained a essential factor of global cardiovascular incidence and mortality, with a rapidly increasing prevalence.^5^ In the United States, women of all ages exhibited higher mortality rates of heart failure compared to men.^6^ Lifestyle factors, environmental exposures, and estrogen-related compounds can induce epigenetic modifications in adult women and developing fetuses/offspring, which can be transmitted through germlines and affect heart failure development. Cancer also exhibited sex differences, with men generally facing a significantly higher cancer risk and mortality compared to women.^3^ Smoking, alcohol consumption, and other lifestyle habits closely associated with cancer, along with variations in gene expression and hormone levels, which resulted in sex disparities of cancer risk. Numerous metabolic processes (including glucose and lipid metabolism), immune system renewal, and nervous system development experienced distinct biological processes in the evolution and development of the human body, which were different between males and females. Such sex differences regulated homeostasis through the activation of sex-determining genes and fetal hormone programming, having significant influence on disease risk.^7^ Currently, most drugs and clinical treatments lack research data on sex differences. However, with advancements in modern medicine, clinical experts increasingly recognize the value of personalized drug delivery plans and precision medicine. Sex differences in various diseases serve as crucial factors to consider when formulating personalized treatment strategies for patients. Therefore, identifying subtle distinctions in different body systems under varying sex statuses can contribute to reveal the potential mechanisms underlying cardiac function, tumors, metabolic disorders, immune-related conditions, and neurological diseases within different populations, thereby further improving clinical disease prognosis (Table 1).

**Table 1:** Clinical trials in sex-related human diseases

Clinical phenotype	Study population	Study objective	Main findings	References
Thyroid hormone and sex-related cardiovascular dysfunction	55031 hospitalized patients with congestive heart failure	Hospitalization time and mortality rate for heart failure	The proportion of women in congestive heart failure inpatients with hyperthyroidism was higher, with a lower median age and longer hospital stay	^20^
Thyroid hormone and sex-related cardiovascular dysfunction	2225 patients with ischemic or non ischemic heart failure	The prognostic impact of thyroid abnormalities on heart failure patients	In patients with heart failure, hypothyroidism is more common in older adults and women. Thyroid dysfunction was significantly associated with increased risk of death in patients with symptomatic heart failure and ejection fraction ≤ 35%	^2^
Hormonal disturbances of Perinatal cardiomyopathy and sex-related cardiovascular dysfunction	44 women with perinatal cardiomyopathy	The impact of subsequent pregnancy on cardiac function in women with perinatal cardiomyopathy	Subsequent pregnancies in women with a history of perinatal cardiomyopathy were associated with significantly reduced left ventricular function and can lead to clinical deterioration and even death.	^27^
Hormonal disturbances of Perinatal cardiomyopathy and sex-related cardiovascular dysfunction	220 perinatal cardiomyopathy women	Comparing the clinical characteristics, manifestations, and prognosis of perinatal cardiomyopathy between African American and African American women	Young African-American women were more likely to develop PPCM and had poorer cardiac performance and prognosis compared with non-African American women.	^24^
Hormonal disturbances of Perinatal cardiomyopathy and sex-related cardiovascular dysfunction	100 perinatal cardiomyopathy patients followed up for 1 year postpartum	Prospectively evaluate the recovery and clinical outcomes of left ventricular ejection fraction in perinatal cardiomyopathy.	During a one-year follow-up, it was found that 13% of women had significant events or sustained severe cardiomyopathy. Black women have more left ventricular dysfunction at 6 and 12 months before and after childbirth.	^26^
Estrogen and sex-related cardiovascular dysfunction	4441 postmenopausal women	The heterogeneity between menopausal age and incidence rate of heart failure was investigated.	Compared with women with lower body mass index and waist circumference, the risk of developing heart failure significantly increases as obesity worsens, especially among women who experience menopause at the age of 55 or older.	^32^
Estrogen and sex-related cardiovascular dysfunction	Including 1401175 postmenopausal women	Risk of heart failure and atrial fibrillation	Postmenopausal women with a history of premature menopause or early menopause may have an increased risk of developing heart failure and atrial fibrillation. Compared with postmenopausal women aged ≥ 50, postmenopausal women aged 40 to 49 had a higher incidence of heart failure and atrial fibrillation with earlier menopausal age.	^31^
Estrogen and sex-related cardiovascular dysfunction	5629 postmenopausal women without heart failure	Relationship between menopausal age and incidence rate of heart failure	Early menopausal age was associated with a moderate increase in heart failure risk	^30^
Estrogen and sex-related cardiovascular dysfunction	2947 postmenopausal women	The relationship between early menopause (occurring before the age of 45) and menopausal age in postmenopausal women and left ventricular remodeling and heart failure events	Early menopause was associated with higher left ventricular mass to volume ratio, and concentric left ventricular remodeling was more severe. The older the menopausal age, the lower the risk of heart failure.	^29^
Sex hormones and sex-related metabolic diseases	424 adults	sex differences in adipose tissue insulin resistance	Both low testosterone levels in men and higher testosterone levels in women can lead to more severe adipose insulin resistance	^146^
Gene and sex-related metabolic diseases	117 White NIDDM patients with diabetic nephropathy and 125 patients without any nephropathy and NIDDM ≥ 10 years were enrolled at Joslin Diabetes Center	Association between angiotensinogen gene M235T polymorphism and the risk of diabetic nephropathy in patients with non-insulin-dependent diabetes mellitus (NIDDM)	The DNA polymorphism M235T in the angiotensinogen gene was highly expressed in men with NIDDM, which may account for the increased risk of diabetic nephropathy in men with NIDDM, but not in women.	^149^
Gene and sex-related metabolic diseases	3,561 patients with type 1 diabetes were from Denmark, Finland, France and Sweden	Relationship between rs5186 polymorphism of angiotensin type II receptor 1 gene (AGTR1) and diabetic nephropathy.	The AA genotype of the AGTR1 rs5186 polymorphism may be associated with a significantly increased risk of diabetic kidney disease in men, but not in women.	^150^
Gene and sex-related metabolic diseases	525 patients with type 2 diabetes	Association of ACE I/D and AGT M235T polymorphisms with based on sex diabetic nephropathy risk.	Female diabetic carriers of the ACE D allele had a significantly increased risk of developing diabetic nephropathy, while there was no significant effect for male diabetic patients. Neither the AGT TT genotype nor the T allele were associated with the risk of diabetic nephropathy in male or female diabetic patients.	^151^
Gene and sex-related cancer	The study included 231 participants, including 138 patients with colorectal cancer, 55 patients with colorectal adenomas and 38 healthy controls	Sex differences in tumorigenic gene expression characteristics in patients with colorectal tumors	The higher the expression of PD-L1, the lower the risk of men developing proximal colorectal cancer. Elevated dMMR/MSI and EGFR expression may increase a woman’s risk of developing proximal colorectal cancer.	^236,237^
Sex hormones and sex-related autoimmune diseases	T cells were isolated from 22 female SLE patients and 17 control women	The number of estrogen receptor subtypes in T cells was compared and the ability of receptor agonist-specific ligands to activate marker gene expression was measured	ERα and ERβ agonists can also increase the expression of calcineurin and CD154 in T cells of SLE patients and promote T cell activation.	^334^
Sex hormones and sex-related autoimmune diseases	The 20 patients with SLE included 7 men and 13 women	To investigate the effects of estrogen in vitro on the production of anti-dsDNA antibodies and total IgG in peripheral blood mononuclear cells in patients with SLE	Estradiol use increased the production of anti-dsDNA antibodies and IgG in patients with active SLE, but not in patients with inactive SLE and the normal population.	^330^
Sex chromosome and sex-related autoimmune diseases	2826 SLE patients, 1033 SS patients, and 7074 controls	Correlation between dose effects of X chromosome and autoimmune disease	The estimated prevalence of SLE and SS in women with 47, XXX was ∼2.5 and ∼2.9 times higher, respectively, than that in women with 46, XX and ∼25 and ∼41 times higher, respectively, than that in men with 46, XY.	^357^
Sex chromosome related gene and sex-related autoimmune disease	33 patients with Sjogren’s syndrome and 15 control subjects were enrolled	To investigate the expression of CXCL9, -10, -11, and CXCR3 in the tear film and ocular surface of patients with Sjogren’s syndrome	Expression of CXCL9, -10, -11, and CXCR3 increased in the tear film and ocular surface of patients with Sjögren’s syndrome.	^392^
Sex chromosome related gene and sex-related autoimmune diseases	From 64 patients with SLE, RA, and SS and 17 healthy blood donors	To explore the correlation between CD40-CD40L co-stimulatory pathway and many autoimmune diseases, including SLE, RA and SS	Sera from SLE and SS patients had significantly higher levels of sCD40L compared to sera from healthy control donors. sCD40L was not detected in urine samples of patients with either active or inactive nephritis and in salivary samples from SS patients or normal subjects.	^366^
Sex hormones and sex-related neurodegenerative diseases	A total of 260 PD patients and 308 controls recruited from the Swedish population were genotyped	Potential contribution of genetic variation in the estrogen receptor β gene to the etiology of PD	Genetic variations in the estrogen receptor beta gene may influence the age of onset of PD.	^457^
Sex hormones and sex-related neurodegenerative diseases	394 AD patients were compared with 469 control subjects	To investigate whether nine single nucleotide polymorphisms across the CYP19 gene are associated with AD	Genetic variations in the CYP19 gene, located at 15q21.1, encoding the brain aromatase gene, may alter the risk of Alzheimer’s disease.	^453^
Sex hormones and sex-related neurodegenerative diseases	10,450 U.S. Medicare participants diagnosed with ALS	10,450 U.S. Medicare participants diagnosed with ALS	Tamoxifen was associated with a lower risk of ALS, while testosterone was associated with a higher risk of ALS in women.	^510^
Sex hormones and sex-related neurodegenerative diseases	Data on 15,826 MS patients from 25 countries	The impact of sex on disability accumulation and disease progression was evaluated to determine whether male MS patients have worse clinical outcomes than women	Male relapsing patients accumulate disability more quickly than female patients. In contrast, rates of disability accumulation were similar in men and women with primary progressive MS.	^469^

*ACE* angiotensin-converting enzyme, *AD* Alzheimer’s disease, *AGT* Angiotensinogen, *ALS* amyotrophic lateral sclerosis, *dMMR* deficient mismatch repair, *EGFR* epidermal growth factor receptor, *ER* estrogen receptor, *MS* multiple sclerosis, *MSI* microsatellite instability, *NIDDM* non-insulin-dependent diabetes mellitus, *PD* Parkinson’s disease, *PD-L1* programmed death-ligand 1, *RA* rheumatoid arthritis, *SLE* systemic lupus erythematosus, *SS* Sjogren’s syndrome

## Sex difference in cardiovascular dysfunction

### Introduction

Cardiovascular disease (CVD) is one of the leading causes of morbidity and mortality in Europe, with mortality rates of 40% in men and 49% in women.^8^ Men had a more significant risk of CVD, ischemic heart diseases, and myocardial infarction than women before menopause. In contrast, women’s risk of chronic CVD and ischemic heart disease death rose dramatically after menopause.^9–11^ Pregnancy and childbirth may lead to young female spontaneous coronary dissection and acute myocardial infarction due to increased cardiac stress.^11^ Heart failure has become a principal cause of female morbidity and mortality. It is estimated that one in five women will develop heart failure by the age of over 40 years old.^12^ Women have a similar prevalence of heart failure as males, but their mortality rate is greater.^13^ Male heart failure is typically characterized by heart failure with reduced ejection fraction (HFrEF) [left ventricular ejection fraction(LVEF) ≤ 40%], while female heart failure tends to be more often characterized by either heart failure with preserved ejection fraction (HFpEF) or mild reduction (LVEF 41-49%).^14^^.^Studies have found that the prevalence of heart failure in China has increased since 2017, particularly among women.^15^ Turecamo et al.^16^ indicated that rural women were at a higher risk of developing heart failure among low-income populations in the southeastern United States. Mansur et al.^17^ discovered that women had a better prognosis in HFrEF than men, while mortality rates in HFmrEF and HFpEF were comparable between male and female. An extensive national inpatient sample research discovered that inpatient death rates were greater among cardiac arrest patients. The prevalence of cardiac arrest among heart failure patients is higher in individuals under 65 years old who have concomitant renal disease and coronary artery disease, whereas it is lower in women or HFpEF patients.^18^

Cardiovascular disease is the most common complication for hyperthyroidism patients. 6% of patients with hyperthyroidism’s initial clinical manifestation are congestive heart failure.^19^ Udani et al.^20^ discovered that congestive heart failure and hyperthyroidism were more common in female patients with a lower median age, who had longer hospital stays. It has been reported that about one-third of patients could have hyperthyroidism cardiomyopathy, resulting in reduced cardiac function.^21^ Hypothyroidism is more common in older people and women heart failure patients. Thyroid dysfunction is related to significantly increased death risk in symptomatic heart failure and LVEF ≤ 35% patients.^2^ The prevalence of pregnancy-related HFpEF has been rising during the last few decades.^22^ Peripartum cardiomyopathy (PPCM) is an idiopathic cardiomyopathy (LVEF < 45%, with or without left ventricular dilatation) that develops during the third trimester of pregnancy or within 5 months after delivery.^23^ PPCM is the most prevalent cardiomyopathy during pregnancy.^23^ Studies have indicated that patients with PPCM in the late postpartum period were at higher risk for more severe heart failure.^24^ In a large community retrospective cohort study, PPCM was responsible for approximately 68% of heart failure causes in pregnant women, resulting in adverse fetal outcomes.^25^ A prospective cohort of PPCM found that even though most women had improved left ventricular ejection fraction at 6 or 12 months of follow-up, 13% of female patients may still appear major events or persistent severe cardiomyopathy.^26^ Women with prior PPCM, particularly those with chronic left ventricular dysfunction, could cause a higher unfavorable pregnancy outcomes. In a cohort study of PPCM pregnant women, 44% of those with left ventricular residual dysfunction gave rise to heart failure clinical manifestations during their second pregnancy. However, those women who recovered from PPCM could still generate systolic dysfunction in later pregnancies.^27^

Estrogen insufficiency is regarded as a major risk factor for women’s cardiovascular disease. Menopause usually occurs in women between the ages of 47 and 51. Epidemiological studies show that postmenopausal women are more likely to cause cardiovascular disease than premenopausal women. This risk is positively correlated with increasing age after menopause. Furthermore, premenopausal women are at a lower risk of developing heart disease than men of the same age, which may be due to the protective effect of estrogen.^28^ Early menopause was linked to a greater left ventricular mass-to-volume ratio and more concentric left ventricular remodeling in a multi-ethnic study of atherosclerosis.^29^ Appiah et al.^30^ indicated that women in early menopause are more susceptible to develop heart failure compared to women in late menopause.^30^ Compared with menopausal women aged ≥50 years, the earlier the age of menopause in menopausal women aged 40-49 years, the higher the incidence of heart failure and atrial fibrillation.^31^ The Atherosclerosis Risk in Communities study found that the worsening of female obesity was relate to significantly increased heart failure risk, especially those who had gone through menopause at age 55 or older.^32^

### Mechanism

**Fig. 1:**
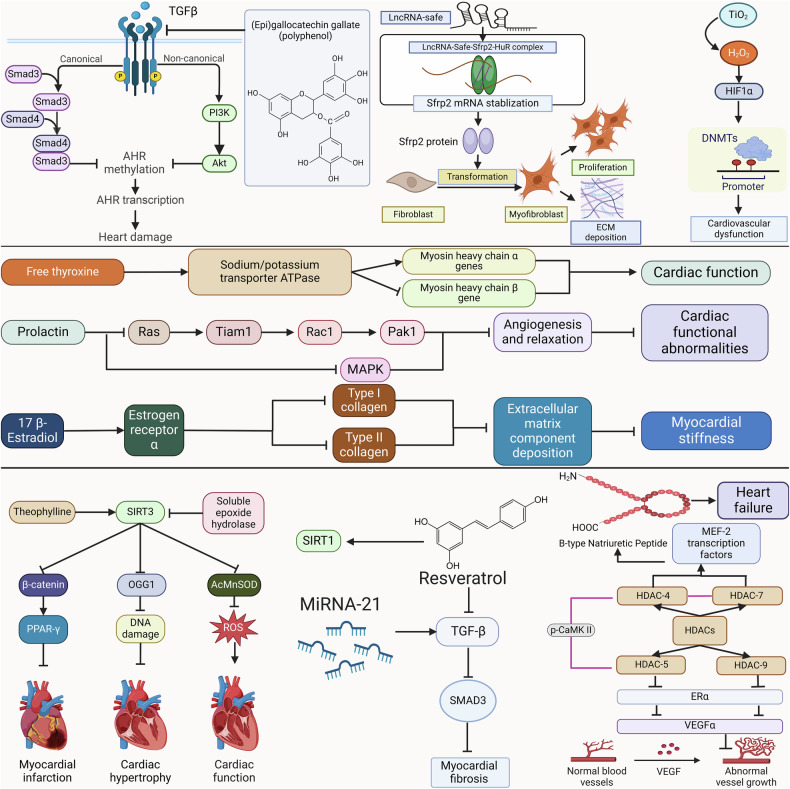
Sex-related mechanisms of cardiovascular dysfunction mainly include hormone and epigenetics regulation. Thyroid hormone can lead to cardiovascular dysfunction by up-regulating the expression of genes encoding sodium/potassium transporter ATPase. Prolactin blocked the mitogen-activated protein kinase activation, accelerating impaired cardiovascular function. E2 down-regulated the type I and III collagen gene expression, promoting myocardial stiffness. DNA methylation, histone modification, and non-coding RNA regulation lead to sex differences in cardiovascular pathophysiology and cardiac functional performance in individuals and offspring. This figure was created with the aid of BioRender (https://biorender.com/). AHR aromatic hydrocarbon receptor, Akt protein kinase B, ECM extracellular matrix, ERα estrogen receptor α, HDAC histone deacetylase, HIF-1a hypoxia-inducible factor-1α, PI3K phosphoinositide 3-kinase, OGG1 oxoguanine-DNA glycosylase-1, PPAR-γ peroxisome proliferator-activated receptor-γ, ROS reactive oxygen species, SIRT Sirtuin, SMAD3 Smad family member 3, TGFβ transforming growth factor β, VEGF vascular endothelial growth factor

Female cardiovascular dysfunction is distinguished from male cardiovascular dysfunction by an imbalance of thyroid hormone metabolism, hormonal alterations in prenatal cardiomyopathy, and changes in estrogen associated with menopause. Furthermore, genetic and environmental factors may influence epigenetic modification in sex-specific ways, resulting in disparities in cardiovascular pathophysiology and heart functional performance. (Fig. 1).

#### Hormone regulation

Plasma or tissue thyroid hormone levels may have some effect on cardiovascular function. Free thyroid hormone increased cardiac β receptor sensitivity to catecholamine, upregulated gene expression for sodium/potassium transporter ATPase, resulting in increased cardiac contractility and cardiac output.^33^ Conversely, thyroid hormone activated the phosphatidylinositol 3-kinase/serine/threonine protein kinase signaling pathway, generating endothelial nitric oxide and vasodilation.^34^ Thyroid hormone induced smooth muscle relaxation by increasing calcium reuptake in the arterioles. They also increased calcium ATPase protein in the sarcoplasmic reticulum and downregulated the phospholamban transcription, thereby causing peripheral vascular dilation and reducing systemic vascular resistance.^33^ Furthermore, thyroid hormone can activate the reninogen angiotensin aldosterone system, aggravating Na+, fluid retention, and cardiac preload. Hyperthyroidism can cause decompensation following high cardiac output and consequent heart failure via various pathways. Hypothyroidism caused damage to ventricular and atrial filling and relaxation, decreased heart rate and myocardial contractility, and increased systemic vascular resistance and artery stiffness, resulting in reduced cardiac output and increased cardiac afterload, exacerbating cardiac dysfunction symptoms.^35^ Subclinical hypothyroidism increased systemic vascular resistance and arterial stiffness by inhibiting vascular smooth muscle cell relaxation and decreasing nitric oxide availability, resulting in impaired ventricular filling and diastolic dysfunction. These effects were reversible with thyroid hormone replacement.^36^ Low T3 syndrome could be caused by type 3 deiodinase activation, which was characterized by decreased T3 and elevated rT3 levels.^37^ Type 3 deiodinase can specifically reduce the thyroid hormone signal transduction in the heart tissue, thereby regulating thyroid function and accelerating cardiac dysfunction progression.^37^ Even if thyroid stimulating hormone was maintained at normal levels, low T3 may increase mortality in heart failure patients.^38,39^

The pathophysiological mechanisms of PPCM are still being investigated. It may primarily induce cardiovascular dysfunction through mechanisms such as anti-angiogenicity, cell apoptosis, inflammation, and immunological response. Hormone secretion during pregnancy had a significant effect on cardiovascular dysfunction occurrence. The adenohypophysis secreted substantial amounts of prolactin (PRL), a polypeptide hormone during pregnancy. In PPCM, oxidative stress imbalance led to the increase of protease cathepsin D and metalloproteinase activities. Proteases can cleave PRL into 16-kDa PRL.^40^ The 16 kDa PRL fragment was biologically active and increased in PPCM patients’ serum. In PPCM, 16 kDa PRL primarily caused heart failure by blocking angiogenesis and promoting cell apoptosis. 16 kDa PRL blocked the activation of the mitogen-activated protein kinase (MAPK) and Ras/Tiam 1/Rac 1/Pak 1 signaling pathways, preventing angiogenesis and relaxation, resulting in cardiac structural and functional abnormalities as well as PPCM occurrence.^41^ Furthermore, 16 kDa PRL stimulated microRNA-146a expression, which decreased neuroblastoma RAS viral oncogene homolog expression and subsequently altered endothelial cell proliferation and metabolism. 16 kDa PRL promoted plasminogen activator inhibitor-1, which blocked urokinase plasmin activator, decreasing endothelial cell migration, extracellular matrix remodeling, and breakdown.^42^ 16 kDa PRL can also reduce the effects of nitric oxide on vascular relaxation and remodeling by inhibiting endothelial nitric oxide synthase and inducible nitric oxide synthase expression.^43^ During the middle and late stages of pregnancy, the placenta released a substantial number of soluble forms, such as tyrosine kinase-1 (sFlt-1). sFlt-1, an anti-angiogenic factor, antagonized angiogenesis, weakened oxidative stress protection and elevated the level of the 16 kDa PRL fragment, all of which had cardiotoxic effects.^44^ The processes mentioned above confirmed the anti-vascular action of 16 kDa PRL and sFlt-1, decreasing heart vascular density. The 16 kDa PRL protein activated nuclear factor κB, stimulated DNA fragmentation via caspases 8, 9, and 3, and initiated both endogenous and external apoptotic pathways.^45^ In addition, 16 kDa PRL prompted endothelial cells to convert miRNA-146a into exosomes, which were taken up by myocardial cells, inhibiting the ErbB pathway and increasing myocardial cell apoptosis.^46^ Persistent inflammatory response could cause cardiac dysfunction in PPCM patients. PRL and interferon γ activated protein kinase B (Akt) and increased the chemokine C-C motif chemokine ligand 2 production, leading to heart inflammation. Akt activation decreased SOD2 expression, lowered antioxidant defense, and exacerbated heart inflammation and fibrosis.^47^ Signal transduction and activator of transcription 3 (STAT3) participated in PPCM development. STAT3 was activated by the production of cytokines (interleukin-6 and chemokine C-C motif chemokine ligand 2) and cell adhesion molecule-1, promoting cardiac cells inflammatory response.^48^ In terms of immune response, β1-adrenaline receptor antibody inhibited peroxisome proliferator-activated receptor gamma coactivator 1 alpha and vascular endothelial growth factor expression to increase myocardial cell apoptosis.^49^ It also played a crucial part in the mechanism of PPCM-induced cardiovascular dysfunction.

Recent research indicates that estrogen protected against sex-related cardiovascular dysfunction by regulating the renin-angiotensin-aldosterone system, oxidative stress, extracellular matrix, inflammatory response, and cell death. 17 β-Estradiol (E2) is the primary female steroid hormone and source of circulating estrogen. In premenopausal women, the ovaries produce and secrete E2. E2 has been shown to reduce angiotensin-converting enzyme activity, angiotensin II levels, tissue responsiveness to angiotensin II, inhibit β-myosin heavy chain expression, calcineurin activity, and activation of extracellular signal-regulated kinase and MAPK, preventing cardiomyocyte hypertrophy.^50^ In terms of the oxidative stress mechanism, the study discovered that E2 boosted angiotensinogen levels and angiotensin-(1-7) synthesis in tissues and circulation, reducing oxidative stress and improving endothelial function.^51^ E2 inhibited p38α-MAPK and increased p38β MAPK activity, reducing reactive oxygen species (ROS) generation in cardiomyocytes and improving survival following ischemia-reperfusion injury.^52^ Furthermore, E2 prevented mitochondrial ROS generation while directly stimulating nitric oxide release by endothelial nitric oxide synthases, thus increasing vasodilation. The study demonstrated that E2, mediated by estrogen receptor (ER)α, down-regulated type I and III collagen gene expression in female cardiac fibroblasts, resulting in lower extracellular matrix component deposition and myocardial stiffness.^53^ E2 inhibited the expression of inflammatory cytokines (interleukin-1β, interleukin-6, and tumor necrosis factor-α) in cardiomyocytes, as well as the levels of inflammatory soluble intercellular adhesion molecule-1, vascular cell adhesion molecule-1, and e-selectin, by activating the ER.^54,55^ As a result, vascular smooth muscle cells and cardiomyocytes’ inflammatory responses were reduced, while cardiomyocyte stiffness, myocardial hypertrophy, and fibrosis were alleviated. In the regulation of the apoptosis mechanism, E2 increased phosphoinositol 3 kinase/Akt signaling and reduced apoptosis-regulated signal kinase-1 and caspase-3-like activity, resulting in decreased cardiac cell death and preventing congestive heart failure in mice.^56^ E2 suppressed p38-MAPK phosphorylation and enhanced atrial natriuretic peptide production by binding to ERβ, and then inhibited ventricular hypertrophy.^57^ E2 can promote atrial natriuretic peptide and brain natriuretic peptide synthesis in cardiomyocytes, reducing phenylephrine-induced hypertrophy.^58^ E2 inhibited the calcineurin/NF-AT3 signaling pathway, increased the phosphoinositol 3 kinase/Akt pathway, and lowered peripheral artery resistance and cardiac hypertrophy, thus protecting the heart.^32,59^ Therefore, E2 inhibited cardiovascular dysfunction development via the mechanisms mentioned above.

#### Epigenetic modification

Many investigations have discovered that epigenetic modification is vital in the pathophysiology of sex-related cardiovascular dysfunction. Cardiomyocytes express sex hormone receptors and respond to sex hormones. Sex hormones regulate heart remodeling and function through DNA methylation. Sebag et al. discovered that the heart wall of female mice was thinner and lighter using echocardiography on male and female C57bl6n mice, and there was evident concentric remodeling after long-term gonadectomy. The expression of cardiac calsequestrin 2 (CSQ2) and sodium-calcium exchanger-1 (NCX1) in women’s hearts increased significantly compared to men, and these expressions decreased following ovariectomy. DNA extracted from the hearts of C57bl6n mice revealed distinct cardiac CSQ2 CpG site dynamic DNA methylation. This methylation is affected by sex and hormonal status. Intact female C57bl6n mice showed lower CpG methylation at CpG site 3 of the CSQ2 promoter and increased CSQ2 expression levels. In estrogen-deficient mice, relative to intact female mice, the lower CSQ2 protein in ovariectomized mice is associated with increased DNA methylation of CSQ2 CpG site 3. Male C57bl6n mice demonstrated the opposite behavior. Following castration (CAS), CpG methylation at CpG site 3 was reduced. As a result, investigations have revealed that estrogen and androgen at least partially govern cardiac calcium homeostasis protein expression via DNA methylation changes, which then affect heart structure and function.^60^ However, the role of sex hormone-regulated DNA methylation on cardiac function in vitro is unclear. Clarifying the effects of sex or hormone deprivation on cardiac function in blood vessels may help to understand the mechanisms by which DNA methylation regulates cardiac dysfunction.

Environmental exposures during pregnancy and perinatal periods can give rise to cardiac dysfunction by disrupting the normal epigenetic processes that occur during early development. Kunovac et al. demonstrated that maternal inhalation exposure to nano-TiO2 during pregnancy destroyed the mouse offspring’s heart and mitochondrial function via epigenetic reprogramming. Maternal inhalation exposure to nano-TiO2 increased fetal H2O2 levels, which triggered hypoxia-inducible factor-1α activity, resulting in increased transcription of genes (such as DNA methyltransferase (DNMT)1) and higher protein production. Increasing DNMT1 expression could cause a global or site-specific increase in methylation, up-regulate fibrogenic genes expression and suppress key genes such as GPx4, contributing to the increase of ROS accumulation and cardiovascular dysfunction.^61^ However, the effects of other specific genes methylation expression and regulatory pathways on mitochondrial and cardiac function may require further exploration in the future. The negative consequences of in-utero exposure to the nonsteroidal estrogen diethylstilbestrol are especially pronounced in women. The DNA methylation of the calsequestrin 2 (CASQ2) promoter was found increased in the hearts of female mice treated with diethylstilbestrol. Thus, exposure to diethylstilbestrol during pregnancy altered adult female ventricular DNA methylation, resulting in myocardial hypertrophy and ventricular remodeling.^62^ Svoboda et al. discovered that Pb exposure during gestation and lactation reduced DNA methyltransferase activity and expression in the heart.^63,64^ Acute Pb exposure can disrupt the cardiac calcium signal, resulting in arrhythmia and reduced cardiac contractility.^65–67^ In addition, prenatal exposure to Epigallocatechin-3-gallate(EGCG) gave rise to myocardial fiber loss and cardiac remodeling in offspring. This mechanism may involve epigenetic alteration of linked genes. Prenatal EGCG exposure increased the pik3r1, TGF-β, and SMAD4 promoters methylation level, decreased the ahr methylation level, and inhibited these genes transcription, resulting in cardiac histological damage in later years. These findings indicate that EGCG may need to be taken with caution during pregnancy.^68^ In addition, vascular calcification is one of the critical pathological manifestations of cardiovascular dysfunction. Adeno-associated virus encoding alkB homolog 1 (Alkbh1)-mediated DNA 6 mA demethylation promoted vascular calcification through osteogenic reprogramming in male mice. It provided a potential target molecule for the early diagnosis and drug development of vascular calcification.^69^

Histone methyltransferase Set7 regulates histone methylation, which influences a wide range of physiologic and pathological processes.^70^ Miranda et al.^71^ discovered that Set7 protein levels were higher in the heart of female obesogenic diet mice. Set7 deletion mice did not develop obesity-induced glucose intolerance or decreased heart function recovery following ischemia/reperfusion injury.^71^ Set7 deletion female mice showed no decrease in left ventricular developed pressure, positive first derivative of left ventricular pressure (+dP/dT), or negative first derivative of left ventricular pressure (−dP/dT) during ischemia/reperfusion, unlike wild-type obese female mice. This is because Set7 deletion in female obesogenic diet mice rectified lower Bcl2 levels after heart ischemia/reperfusion, resulting in reduced cardiomyocyte apoptosis and improved cardiac recovery after ischemia/reperfusion.^71^ Further studies are needed to investigate the effect of Set7 in obesity-induced glucose homeostasis and insulin resistance. In addition, the specific mechanism by which Set7 deletion prevents the reduction of cardiac Bcl2 levels in the I/R response of obese female mice needs to be further clarified. KDM5C and histone lysine specific demethylase 5D (KDM5D) genes encode H3K4 histone demethylase on the X and Y chromosomes, respectively. Kosugi et al.^72^ constructed female mice with KDM5C knockouts and male mice with KDM5C and KDM5D knockouts, and discovered that noncompaction cardiomyopathy occurred in both types of mice. However, the mechanism was unclear.^72^ As a muscle-specific histone methyltransferase, Smyd1 participated in regulating cardiac mitochondrial energy.^73^ Oka et al.^74^ demonstrated that pressure overload lowered Smyd1 expression and binding to the H3K4Me3-rich promoter region of Perm1, resulting in the downregulation of Perm1 expression.^74^ The modifications described above decreased enzyme expression and their components that encode the tricarboxylic acid cycle, electron transport chain, mitochondrial fatty acid oxidation, and glucose consumption. As a result, mitochondrial repair was hampered, leading to heart failure.^75^ The class IIa histone deacetylases (HDACs) (HDACs 4, 5, 7 and 9) recruit class I HDACs and interact with other transcriptional repressors to perform their functions.^76^ Rooij et al.^77^ discovered that female knocked-out HDAC5 and HDAC9 mice showed reduced left ventricular remodeling following myocardial infarction. This is due to the decreased levels of HDAC5 and HDAC9, which facilitated the activation of ERα signaling. These alterations boosted the target gene vascular endothelial growth factor α expression and neovascularization in the infarction location, which alleviated left ventricular remodeling following myocardial infarction.^77^ Class II HDACs regulated cardiac remodeling by decreasing the enhancer factor 2 (MEF2) activity.^78,79^ Calmodulin-dependent protein kinase phosphatase (CaMKP) regulates CaMKII-mediated MEF2 activation in a sex-specific way. Pressure overload frequently causes systolic and diastolic dysfunction, which manifests as pathological left ventricular hypertrophy. Pre’vilon et al.^78^ mimicked pressure overload with transverse aortic constriction (TAC) mouse models and discovered that CaMKP were expressed in the male and female mice nucleus and cytoplasm, respectively. Consequently, female mice with nucleus phosphorylated CaMKII escaped dephosphorylation and deactivation by cytoplasmic CaMKP. Phosphorylated-CaMKII increased the output of HDAC4, HDAC5, and HDAC7 from the nucleus, decreasing their inhibitory effect on the activity of MEF2 transcription factors, and promoting pathological left ventricular hypertrophy markers Brain Natriuretic Peptide and α-SK gene transcription, leading to cardiovascular dysfunction.^78^ Further work is needed to identify sex differences in CaMKP expression and related signaling processes in cardiomyocytes and the molecular mechanisms upstream of this sex-specific CaMKP compartmentalization after TAC. Sirtuins 1 and 3 have critical roles in the pathophysiology of sex-related cardiovascular dysfunction. Garcia et al.^80^ discovered that rats missing methyl donors had a lower ratio of S-adenosylmethionine (SAM) to S-adenosylhomocysteine (SAH) during pregnancy and lactation. Peroxisome proliferator-activated receptor-γ coactivator-1α (PGC-1α) activation is dependent on protein arginine methyltransferase-1 (PRMT1) methylation and sirtuin 1 deacetylation. Decreased sirtuin1 and PRMT1 expression led to an imbalance in PGC-1α acetylation/methylation. In rats lacking methyl donors, downregulation of PGC-1α, PPARα, and estrogen-related receptor α in the myocardium decreased the expression of mitochondrial enzymes and respiratory complexes I and II involved in fatty acid oxidation. This led to myocardial hypertrophy and mitochondrial arrangement disorder.^80^ Hajializadeh et al.^81^ showed that estrogen can lower oxidative stress, apoptosis, and inflammation by increasing sirtuin 1 level, thus delaying pathological cardiac hypertrophy and heart failure occurrence. Doxorubicin, the most often used chemotherapeutic medication for female breast cancer patients, frequently reduces clinical efficacy due to cardiotoxicity.^81^ Anthracycline cardiomyopathy is caused by oxidative stress, mitochondrial damage, apoptosis, and cardiac fibrosis. Activation of sirtuin 1 and sirtuin 3 reduced doxorubicin-induced cardiotoxicity. Cappetta et al.^82^ discovered that doxorubicin caused heart systolic and diastolic dysfunction by increasing fibroblast activity and collagen deposition. Resveratrol, a sirtuin 1 activator, improved heart function by decreasing TGF-β and pSMAD3/SMAD3 levels, fibroblast activation, and doxorubicin-induced cardiac fibrosis. Pillai et al.^83^ demonstrated that doxorubicin therapy lowered sirtuin 3 levels in cardiomyocytes while increasing mitochondrial protein acetylation and ROS generation, resulting in mitochondrial breakdown, cell death, and cardiac cytotoxicity. Sirtuin 3 transgenic mice reduced doxorubicin-induced cardiac hypertrophy by decreasing oxygen-guanine-DNA glycosylase-1 levels and protecting mitochondrial DNA. Song et al.^84^ found that the ejection fraction and fraction shortening were significantly reduced and myocardial fibrosis and myocardial apoptosis were significantly aggravated in estrogen-deficient female C57BL/6 mice at 28 days after infarction. Myocardial cells at the periphery of myocardial infarction showed decreased expression of sirtuin 3 and peroxisome proliferator-activated receptor γ (PPARγ), but dramatically enhanced expression of β-catenin. β-catenin, a critical factor in the Wnt and TGF-β1-SMAD-3 signaling pathways, increased myocardial fibroblast transformation, resulting in myocardial fibrosis.^85^ PPARγ belongs to a nuclear steroid receptor superfamily that requires ligands to function. Activating PPARγ reduced the size of myocardial infarctions in rats with acute ischemia-reperfusion damage.^86^ Sirtuin 3 activator theacrine reduced post-myocardial infarction cardiac remodeling induced by myocardial fibrosis and apoptosis. The expression of sirtuin 3 protein and PPARγ was dramatically increased. β-catenin expression was dramatically reduced in myocardial cells along the border of myocardial infarction. Studies indicate that Sirtuin 3 activator theacrine improved heart function after estrogen-deficient mice myocardial infarction by boosting sirtuin 3 levels and modulating the β-catenin/PPARγ signaling pathway. However, the effect of theacrine in postmenopausal women coronary heart disease and myocardial infarction prevention or treatment was unclear and needed to be confirmed in clinical trials.^84^ In addition, intrauterine growth restriction is an obstetric complication characterized by placental insufficiency and subsequent cardiovascular remodeling, which can progress to adult cardiomyopathy. Jamieson et al.^87^ discovered that compared to old male soluble epoxide hydrolase (sEH) knockout mice, old female sEH knockout animals showed improved contractile performance and less cardiac hypertrophy. Old female sEH knockout mice protected the heart by keeping greater sirtuin 3 activity, lowering AcMnSOD levels and oxidative stress, and preserving normal mitochondrial ultrastructure.^87^ Sirtuin 6 can maintain the homeostasis function of endothelial cells, delay vascular senescence and prevent cardiomyocyte hypertrophy. Study found that sirtuin 6 in male mice degraded runt-related transcription factor 2 by deacetylation and ubiquitination to slow the VSMC osteogenic differentiation and calcification.^88^ In male mice, sirtuin 6 inhibited GATA6 transcription by deacetylating Nkx2.5. GATA6 inhibited DNA damage repair, thereby accelerating VSMC osteogenic phenotype transformation and calcification.^89^ However, capsaicin led to increased expression of sirtuin 6, which in turn promoted sirtuin 6-mediated hypoxic-inducible factor-1α deacetylation and degradation to play a protective role in vascular calcification.^90^

Lactate was found to be an epigenetic modifying factor. Hypoxia-induced lactate generation has been demonstrated to increase histone lysine lactylation in gene promoter areas, which regulates gene expression.^91^ Li et al.^92^ discovered increased lactate levels and histone lactylation in the preeclampsia placenta. Only the fibrosis-related genes FN1 and SERPINE1 were elevated in the preeclampsia placenta, as well as the HTR8/SVneo and TEV-1 cell lines. Upregulation of the FN1 and SERPINE1 genes was linked to decreased cardiac function and heart failure development.^93–95^ The study found that 1% O2 and sodium lactate treatment dramatically enhanced H3K18 lactylation levels in the FN1 and SERPINE1 promoter regions of HTR8/SVneo and TEV-1 cells. Furthermore, the addition of the lactate dehydrogenase inhibitor oxalate decreased the elevated FN1 and SERPINE1 gene promoter regions H3K18 lactylation levels caused by 1% O2. As a result, the study revealed that lactate, as an epigenetic regulatory factor, enhanced the FN1 and SERPINE1 expression via histone lactylation under hypoxia. This demonstrates the potential effect of epigenetic changes mediated by glycolytic metabolic intermediates in preeclampsia pathogenesis.^92^

Non-coding RNA has emerged as a crucial regulator of heart disease, with the potential to be a therapeutic target for sex-related cardiovascular dysfunction. MiRNA may have opposite effects on cardiac structure and function in different sexes. García et al. discovered that miRNA-29b expression was increased in the left ventricular myocardium of transverse aortic contraction female mice under pressure stress, but down-regulated in the transverse aortic contraction male animals. MiRNA-29b targets (collagen and GSK-3β) showed that males had more severe myocardial fibrosis, whereas females had greater hypertrophy. The cardiac systolic and diastolic function of transverse aortic contraction females was worse, indicating that miRNA-29b had a negative effect on female hearts but a positive effect on male left ventricles during pressure overload.^96^ Florijn et al. found low miRNA-34a, miRNA-224, and miRNA-452 levels in diabetic patients with left ventricular diastolic dysfunction and female diabetes patients with estimated glomerular filtration rate < 60 ml/min. Compared with men, diabetic HFpEF women had higher angiopoietin-2 expression and the miRNA-224/452 cluster on the X chromosome. The increased plasma angiopoietin-2 level suggested that HFpEF women were predisposed to microvascular injury, which was consistent with previous research demonstrating that coronary microvascular dysfunction was a distinct pathophysiology of HFpEF women.^97,98^ Furthermore, HFpEF women had considerably greater plasma levels of miRNA-224 and miRNA-452 than males. This could be because the X chromosomal origin of both miRNAs was only related to female-specific differential expression.^99^ The obesity diet elevated the expression of miRNAs associated with heart hypertrophy in female mice. Oliveira Silva et al. discovered that the amount of miRNA-143-3p in the obese female mice hearts increased, which was accompanied by a drop in Sox6 mRNA levels and increased MYH7 expression. Loss-of-function experiments in cardiomyocytes found that inhibiting miRNA-143-3p increased Sox6 mRNA expression while decreasing Myh7 levels. As a result, the miRNA-143-3p-Sox6-Myh7 pathway could be the primary mechanism causing heart hypertrophy in obese female mice. Further studies are needed in the future aimed at determining sex differences in the miRNA-143-3p-Sox6-Myh7 axis in human heart tissues and the effects on heart structure, which may provide some enlightenment and theoretical basis for the development of sex-specific cardio-targeted miRNA-143-3p therapies.^100^ LncRNA Myosin Heavy Chain Associated RNA Transcripts (MHRT) have recently been discovered to be cardioprotective lncRNAs. Zhang et al. discovered that rs3729829 locus allele (GA/AA) carriers of the MHRT gene showed a significantly higher congestive heart failure risk than GG genotype carriers in young and female patients and those without hypertension and diabetes, implying that detecting MHRT gene rs3729829 in these subpopulations may be more clinically significant than in older and male patients, as well as those with hypertension and diabetes.^101^ Meessen et al. discovered that in heart failure patients with a higher NYHA class, worse renal function, and lower hemoglobin levels, circulating long non-coding RNA LIPCAR levels were greater, and these relationships were stronger in females than in males.^102^ Zhuang et al. performed experimental and bioinformatic investigations of mouse and human heart tissues and cells to confirm that the lncRNA OIP5-AS1 was cardiomyo-rich. Female mice deficient in lncRNAs OIP5-AS1 exhibited progressive heart failure following cardiac pressure overload (TAC), whereas male mice did not. Female OIP5-AS1 knockout mice’s loss of mitochondrial transcription factors PGC1α and Esrrg (ERRgamma) after TAC may impair their ability to generate energy, leading to cardiac systole dysfunction and increased heart failure risk. As a result, the study revealed that lncRNAs OIP5-AS1 could be related to mitochondrial function and heart failure development sex-specific changes.^103^

As a common manifestation of various heart diseases, cardiac fibrosis eventually gave rise to end-stage cardiovascular dysfunction. Non-coding RNA is critical in regulating both normal and pathological cellular processes, including gene expression programs linked to sex-based cardiovascular dysfunction. Zhang et al. revealed that miRNA-21 activated the TGF-β/Smad2/3 signal pathway and efficiently inhibited cardiac fibrosis by inhibiting it. Estrogen has an antifibrotic action. Men had higher gene expression levels of the TGF-β signaling pathway than women but lacked estrogen’s protective impact. It was believed that activating the male TGF-β signaling pathway could enhance heart fibrosis and dilated cardiomyopathy development.^104^ Hao et al. discovered that lncRNA-Safe was significantly expressed in female mice fibroblasts and fibrous tissue following myocardial infarction. LncRNA-Safe exacerbated cardiac fibrosis, at least in part, by increasing Safe-Sfrp2-HuR complex-mediated Sfrp2 mRNA stability and protein expression. LncRNA-Safe knockdown prevented TGF-β-induced cardiac fibrosis and improved cardiac function in female myocardial infarction mice by decreasing fibroblast proliferation, fibroblast-myofibroblast transition, and type I collagen secretion. The data showed that lncRNA-Safe could be a new target for anti-myocardial fibrosis treatment in women.^105^ LINC00707 is a kind of long non-coding RNA that can regulate multiple diseases. Zhao et al. discovered that LINC00707 and S1PR1 expressions were considerably lower in female rheumatic heart disease rats. Female rats with rheumatic heart disease had higher levels of Collagen III/I (COLIII/I), COLIIIα1 mRNA, FSP1 mRNA, and miR-145-5p in their heart valve tissues. The luciferase reporter experiment confirmed that miR-145-5p was directly regulated by LINC00707. LINC00707 dramatically reduced the miRNA-145-5p expression. S1PR1 is a downstream gene of miRNA-145-5p, which is negatively regulated. Therefore, LINC00707 can reduce myocardial damage and fibrosis in female rheumatic heart disease rats via modulating miRNA-145-5p/S1PR1. Future studies need to further explore the potential mechanisms between S1PR1, miR-145-5p, LINC00707 and rheumatic heart disease.^106^ Guo et al. identified the effect of the lncRNA RASSF1-AS1 in cardiac fibrosis development. RASSF1-AS1 expression was increased during the cardiac fibrosis process in female mice. Over-expression and knockout experiments of female mice primary cardiac fibroblasts found that RASSF1-AS1 negatively regulated the RASSF1A protein level. RASSF1-AS1 aggravated cardiac fibrosis by directly binding to RASSF1A mRNA and inhibiting its translation. Therefore, RASSF1-AS1 may be a potential therapeutic target for cardiovascular dysfunction.^107^ In conclusion, miRNA-21, lncRNA-Safe, LINC00707, lncRNAs OIP5-AS1 and RASSF1-AS1 can be used as new therapeutic targets related to sex-related cardiovascular dysfunction in the future.^103–107^ Therefore, the regulation of non-coding RNA changes has important clinical significance for the clinical diagnosis and prognosis of sex-related cardiovascular dysfunction. At present, these epigenetic targets or drugs have not been used in sex-related cardiovascular dysfunction clinical practice. However, we believe that through continuous exploration and large-scale clinical studies in the future, more emerging epigenetic drugs for the treatment of sex-related cardiovascular dysfunction will be created in order to better improve cardiovascular disease patients’ symptoms and prognosis.

### Clinical implications

Sex-related cardiovascular dysfunction treatment aim to improve symptoms, slow or reverse cardiac deterioration, and reduce readmission and mortality. The treatment of cardiovascular dysfunction mainly includes life intervention, drug therapy and interventional therapy. In addition, we also need to timely risk factor intervention for diseases such as PPCM and estrogen deficiency that can cause female cardiac dysfunction, so as to better improve cardiovascular prognosis.

In order to decrease cardiac dysfunction incidence rate caused by PPCM, it is necessary to take preventive strategies of healthy diet and exercise to improve the female cardiovascular health of childbearing age before pregnancy. Acute PPCM patients require coordinated cardiovascular and obstetric care to ensure the mother and baby’s health and safety during pregnancy. The 2018 ESC guidelines introduced the concept of “BOARD” for gestational heart failure treatment, which may benefit a large number of PPCM patients. Doctors should pay attention to contraindicated drugs, such as angiotensin converting enzyme inhibitors, angiotensin receptor blockers, warfarin during PPCM treatment.^108^ Most importantly, bromocriptine should be used in conjunction with prophylactic doses of anticoagulants such as heparin to reduce the thromboembolism risk.^109^ Furthermore, numerous studies have advised bromocriptine in conjunction with regular heart failure medication as a specialized treatment regimen for severe PPCM. In female patients with PPCM XI related cardiogenic shock and an initial average left ventricular ejection fraction of 15%, temporary percutaneous mechanical circulation support using Impella heart pumps contribute to improve heart failure symptoms.^110^ Pentoxifylline combination with other heart failure drugs contribute to improve PPCM patients prognosis.^111^ Therefore, clinicians should focus on PPCM management and therapy, which is critical for preventing and delaying cardiovascular dysfunction in pregnant women, as well as lowering the high maternal and fetal mortality rates.

To prevent and treat cardiovascular dysfunction in menopausal women, it is recommended that they engage in frequent moderate exercise and maintain a healthy weight and waist size.^112^ Furthermore, it is better that hormone replacement therapy should be started as early as possible in the first year after menopause in postmenopausal women to decrease cardiovascular risk.^113^ The 2022 North American Menopause Association Hormone Therapy Position Statement emphasizes that the benefits will far outweigh the risks for women who start hormone replacement therapy at age <60 years or within 10 years of menopause.^113^ Current hormone replacement therapy medication regimens include estrogen alone or with progestogen, topical estrogen usage, the estrogen/progesterone cycle sequence, and a continuous estrogen/progestogen combination. At present, most of the estrogens supplemented in clinical practice are natural estrogens, such as estradiol valerate and 17β-hydroxysteroids, which can avoid the endometrial cancer additional risk as much as possible. In addition, women with uterus need to combine with progestogens for hormone replacement therapy. Progesterone, dydrogesterone and other progesterone with relatively small risk of endometrial cancer are recommended to reduce endometrial cancer risk. Continuous combination regimens are the most effective for preventing endometrial hyperplasia and cancer. Oral therapy is recommended for young, newly menopausal patients with obvious symptoms who need hormone replacement therapy to work quickly. Percutaneous therapy is suggested to decrease thrombosis risk for patients with prolonged menopause and insignificant symptoms.^113^

At present, there is no sex specificity in the diagnosis and treatment of cardiovascular dysfunction guidelines in China, Europe, and America. Although women are underrepresented in some studies and there is not much prospective randomized data on the safety and efficacy of sex-specific treatments, some meaningful results have been obtained from some studies. Previous studies have found that sacubitril/valsartan can improve heart function and reduce hospitalization rates in female heart failure patients with preserved ejection fraction.^114^ As for β receptor blockers, carvedilol has more benefits in treating male heart failure, while metoprolol improves heart failure better in women.^115^ There is no sex difference in the treatment of heart failure with aldosterone receptor antagonists, sodium glucose cotransporter 2 inhibitors, ivabradine, and soluble guanylate cyclase agonists. Cardiac resynchronization therapy benefits women with heart failure more than men.^116^ In REPORT-HF clinical studies, women have a higher risk of heart failure with preserved ejection fraction and its complications compared with men. Women with acute heart failure and reduced left ventricular ejection fraction received lower frequency of coronary angiography, cardiac stress testing, and coronary revascularization treatment than men, and these sex differences were not correlated with national income levels and geography.^117^ In the clinical trial cohort of contemporary high-risk myocardial infarction patients, women with acute myocardial infarction and decreased left ventricular ejection fraction had a higher risk of heart failure hospitalization and comorbidities. Sacubitril/valsartan had the same good therapeutic impact and tolerability as ramipril, regardless of sex.^118^ Therefore, when patients’ clinical indications match the appropriate conditions for sacubitril/valsartan and there are no adverse events, clinical doctors should advise heart failure patients to prioritize sacubitril/valsartan treatment. The post-hoc analysis of the VICTORIA trial (Vericiguat Global Study) revealed that the therapeutic benefit of vericiguat on heart failure patients with decreased ejection fraction was unaffected by sex differences.^119^ In the GALACTIC-HF clinical trial, males and females showed no significant difference in the therapeutic impact of omecamtiv mecarbil on heart failure patients with decreased ejection fraction. This could be attributed to the trial’s lower female participation rate (women make up only 21% of GALACTIC-HF participants).^120^ The study discovered that women’s plasma concentrations of omecamtiv mecarbil were higher, hence they required a lower dose. Despite the higher plasma omecamtiv mecarbil concentration, omecamtiv mecarbil was well tolerated in women compared with men.^120^ The frequency of ischemia diseases in women was less, resulting in less myocardial scarring and arrhythmia matrix. Omecamtiv mecarbil’s selective impact on myocardial globulin caused greater myocardial contractility in women, resulting in a decreased incidence of major adverse events (ventricular arrhythmias and ischemic events) in women.^121,122^ Currently, the benefits and drawbacks of medicines and surgical surgery for treating cardiovascular dysfunction in women indicate that more clinical trials are required to investigate and validate. To achieve these, it is necessary for women to have sufficient representativeness in clinical research and to prospectively investigate sex differences, which may be of great significance for delaying the occurrence and progression of sex-related cardiovascular dysfunction patients.

## Sex difference in metabolic disorders

### Introduction

In recent years, more evidence indicated that sex differences may participate in the numerous metabolic diseases pathogenesis and treatment. Metabolic homeostasis disorders caused by obesity, metabolic syndrome, insulin resistance, and atherosclerotic dyslipidemia in its development process is due to an intrinsic difference in genotype (i.e. sex chromosome), as well as a significant sex difference in changes in the surrounding metabolic environment (such as sex hormones, other hormones, or metabolites).^123^ Globally, the proportion of overweight and obese women is higher than that of men.^124^ Type 1 diabetes is the only common autoimmune disease not dominated by women.^125^ Girls tend to have more residual β cell function in their bodies than boys. Female estrogen plays an important role in preventing type 1 diabetes in adolescent girls.^126,127^ However, women have a higher risk of developing type 2 diabetes than men.^125^ In obese patients, the effect of insulin on the target organs is reduced, and the nutrient storage capacity is impaired, resulting in insulin resistance and early metabolic dysfunction. Insulin resistance in adipose tissue is the core of metabolic diseases, as adipose tissue is essential for energy regulation.^128^ Metabolic syndrome is a key factor associated with obesity and insulin resistance. Metabolic syndrome pathogenic mechanism was that caused inflammation by decreasing the sensitivity of pro-inflammatory adipokines to insulin in visceral adipose tissue, which leaded to cardiometabolic disorders and increased cardiovascular disease risk.^129,130^ There are sex differences in the metabolic syndrome and insulin resistance development. Males are more susceptible to develop metabolic syndrome than premenopausal females; nevertheless, the protective effect of females gradually decreases as estrogen levels in the body decline. Postmenopausal women and men tend to have a higher risk of developing insulin resistance than premenopausal women.^131^

### Mechanism

**Fig. 2:**
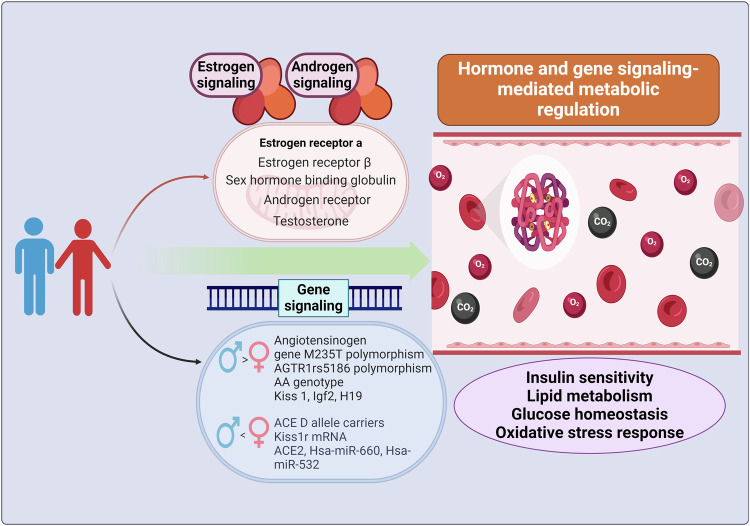
The regulation of sex hormones and gene affects insulin sensitivity, lipid metabolism, glucose homeostasis, and oxidative stress responses. Hormone regulation mainly includes estrogen receptor α, estrogen receptor β, sex hormone binding globulin, androgen receptor and testosterone. In the metabolic diseases regulated by gene signaling, the genes with higher expression in males than in females mainly include Angiotensinogen gene M235T polymorphism, AGTR1rs5186 polymorphism AA genotype, Kiss 1, Igf2 and H19. However, the genes that women expressed more than men mainly included ACE D allele carriers, Kiss1r mRNA, ACE2, Hsa-miR-660 and Hsa-miR-532. Hormonal and gene signaling regulate insulin sensitivity, lipid metabolism, glucose homeostasis, and oxidative stress responses, thereby influencing the occurrence of metabolic diseases. This figure was created with the aid of BioRender (https://biorender.com/). ACE angiotensin-converting enzyme, AGTR1 angiotensin II type 1 receptor gene

It is generally understood that sex disparities exist in the cause, pathophysiology, and treatment effectiveness of metabolic diseases. Hormone, gene and epigenetic regulation have important effects on metabolic homeostasis. Therefore, understanding the potential mechanism differences between male and female metabolic diseases is critical for patients management and treatment. (Fig. 2).

#### Hormone regulation

Hormone plays an important role in sex differences, which are not only related to the adipose tissue anatomical distribution, but also to energy and glucose homeostasis. Therefore, men and women have different susceptibility to metabolic disorders such as insulin resistance and metabolic syndrome. Leptin is a circulating peptide hormone produced by adipocytes. Plasma leptin is related to insulin resistance, inflammation and thus participate in the long-term metabolic diseases regulation. Study has found that plasma leptin levels were higher in women than in men because of their higher proportion of adipose tissue and faster leptin synthesis. In the Jackson Heart Study, it was showed that leptin levels were significantly correlated with female stroke risk, but not in male.^132^ Adiponectin is a kind of cytokine mainly produced by adipose tissue, which can regulate the coronary heart diseases, diabetes, and metabolic disorders pathophysiological process. Women had higher levels of adiponectin than men. Postmenopausal women plasma adiponectin levels were significantly higher compared with premenopausal women. According to the Frachial Offspring study, high adiponectin levels was an important protective factor for preventing the coronary heart disease risk in males. Adiponectin levels were inversely associated with the coronary heart disease risk.^133^

Endogenous estrogen regulated glucose homeostasis by activating ERα and ERβ.^134–136^ The ERα expression in female adipose tissue was higher than that in male adipose tissue. ERα activation enhanced glucose-stimulated insulin biosynthesis, reduced islet toxic lipid accumulation, and promoted β cell survival through pro-apoptotic stimulation. ERβ activation promoted glucose-stimulated insulin secretion.^137^ Estrogen regulated liver insulin production and transport, improves liver insulin response, and reduced liver insulin degradation, thereby lowering blood glucose.^135,136,138^ Estrogen mediated the improvement of insulin sensitivity in adipocytes, protected adipocytes from oxidative stress, and improved insulin stimulated glucose uptake by skeletal muscle.^124^ Estrogen improved heart function through ERα and alleviated myocardial damage induced by insulin resistance. Estrogen can also enhance nitric oxide production in the vascular endothelium and promote vascular relaxation.^135,136^ Furthermore, estrogen signaling also affects lipid metabolism. Bryzgalova et al. found that estrogen increased liver Lepr expression by binding to ERα, upregulated genes involved in lipid transport, thus regulating liver insulin sensitivity and glucose homeostasis.^139^ Taken together, estrogen stimulated insulin secretion and regulated the expression of lipid genes, thereby lowering blood glucose and preventing type 2 diabetes. The discovery could eventually open up new ideas for treating type 2 diabetes. However, the specific mechanism by which estrogen in males promotes insulin transport to the muscle needs to be further investigated in the future.

Androgens play an important role in physiological processes such as metabolic syndrome. Androgens could inhibit fat deposition.^140^ Androgen deficiency could decrease insulin sensitivity in healthy young men.^141^ Lack of androgen receptor (AR) promoted adiponectin secretion and significantly increased triglycerides and decreased insulin sensitivity. Androgens are involved in lipid metabolism regulation. Normal serum levels of androgens had a protective effect on metabolism.^142^ Testosterone levels in obese men were generally low, as were serum total testosterone and free testosterone levels in type 2 diabetic patients.^143^ A 25% decrease in serum testosterone in elderly men can lead to doubling insulin resistance.^144^ Severe testosterone deficiency caused blood lipids changes, thus increasing diabetes and vascular diseases risk. Clinical research has shown that testosterone enhanced insulin sensitivity in men with hypogonadism, as well as decreased the metabolic syndrome and cardiovascular complications risk.^145^ Furthermore, testosterone accelerated insulin resistance in overweight or obese women adipose tissue. Therefore, both males testosterone deficiency and females excessive testosterone can increase the adipose insulin resistance risk.^146^ These studies revealed that hypogonadism was an independent risk factor for metabolic syndrome. The androgen/AR signal is involved in the regulation of cellular insulin signaling, glucose, lipid, and metabolic homeostasis development, and has significant sex differences.^147^ In addition, sex hormone binding globulin (SHBG) contributed to type 2 diabetes patients glucose metabolism and insulin resistance. A meta-analysis of multiple prospective clinical studies revealed that women had higher SHBG levels compare with men. SHBG was an independent risk factor for female type 2 diabetes patients. SHBG concentrations in women were inversely associated with the risk of developing type 2 diabetes.^148^ Therefore, these studies findings may have implications for the broader clinical use of sex-related hormones to assess metabolic disease risk. It also emphasized the importance of considering sex as a key factor in assessing the cardiac and metabolic diseases risk in order to improve their management and prevention.

#### Gene and epigenetic modification

Gene and epigenetic modifications are important for regulating the occurrence of metabolic diseases in both men and women. Joslin Diabetes Center study showed that the DNA polymorphism M235T in the angiotensinogen gene was highly expressed in type 1 diabetes male patients, which increased diabetes nephropathy risk, but no difference was found in females.^149^ In a large cohort study of type 1 diabetes patients from Denmark, Finland, France, and Sweden discovered that the angiotensin II type 1 receptor gene (AGTR1) rs5186 polymorphism AA genotype significantly increased male diabetes nephropathy risk but had no significant impact on women.^150^ Tien et al. found that female diabetes carriers with the angiotensin-converting enzyme (ACE) D allele had a significantly higher risk of progressing to diabetes nephropathy, whereas no significant correlation was observed for men with type 2 diabetes.^151^ Kisspeptin (KP) regulated the hypothalamic pituitary gonadal (HPG) axis. KP was encoded by the KISS 1 gene. The deficiency of KP or its functional receptor KISS 1 R generated hypogonadotropic hypogonadism. Ziarniak et al. discovered that the Kiss1r mRNA expression in the female type 2 diabetes rats liver and adipose tissue increased significantly, whereas Kiss1r expression in male type 2 diabetes rats remained unchanged.^152^ Type 2 male diabetes rats had higher levels of Kiss 1 mRNA in the pancreas and Kiss 1 protein in the liver, whereas type 2 diabetes female rats only had an increase in Kiss 1 mRNA in the liver.^153^ Study has shown that the increase in Kiss 1r mRNA levels in type 2 diabetes female rats was associated with promoter hypermethylation.^152^ Hyperglucagon may increase liver KP. KP decreased glucose stimulated insulin secretion and insulin levels. Therefore, developing drugs that antagonize KP is expected to be a promising therapeutic strategy to enhance type 2 diabetes β cell function.^154^ Ding et al. demonstrated that due to methylation imbalance in the differential methylation region, the expression of imprinted genes Igf2 and H19 in the islets of male gestational diabetes offspring was higher than that in female offspring, potentially leading to ultrastructural defects and functional impairment of the islets.^155^

Sex-based whole genome DNA methylation data of human pancreatic islet clusters revealed that the DNA methylation and gene expression of 61 x chromosome genes and 18 autosomal genes differed between men and women. Among them, six genes including APLN, ATF4, and HMGA1 may be regulated insulin secretion.^156–158^ However, sixteen genes including ARSD, KDM5C, and KIF4A could have sex differences.^159–161^ The CpG islands within the promoter region indicated that the X chromosome methylation level in active females was lower than that in inactive females.^162^ In male pancreatic islets, DNA methylation at specific CpG sites on the X chromosome was higher than in females. The expression of ACE2 encoding angiotensin-I-converting enzyme 2 in the males islets was lower than that of females. Studies have shown that in human islets, the CpG site labeled microRNA had different DNA methylation between sexes, resulting in type 2 diabetes patients islet function changes.^163^ In human islets, DNA methylation affected miRNAs and their target genes expression. There were three miRNAs with significantly differentially expressed sex-based DNA methylation in autosomes: hsa-miR-548H4, hsa-miR-220B and hsa-miR-663B. Sex-based DNA methylation differences were found at 160 miRNA-annotated loci on the X chromosome, with 22 loci showing higher methylation levels in females. Women also had six unique hypermethylated miRNAs. There are 138 loci in males that exhibited a high degree of methylation, corresponding to 59 unique miRNAs with high methylation in males. Compared with males, hsa-miR-660 and hsa-miR-532 located on the X chromosome in female pancreatic islets showed lower DNA methylation levels and higher expression. In females, increased DNA methylation of NKAP and SPESP1 proximal promoter led to decreased expression. Silencing of two X chromosome genes, APLN and NKAP, and an autosomal gene, BCL11A contributed to increased insulin secretion in clone beta cells.^164^ Study revealed that chromosomal range and gene-specific sex differences existed in human pancreatic islet X chromosome DNA methylation, whereas autosomal chromosomes only showed site-specific differences. These epigenetic modifications based on sex-specific metabolic phenotypes influence insulin secretion by regulating differential gene expression and microRNA levels in human pancreatic islets.

### Clinical implications

The presence of metabolic disease risk factor clusters increased cardiovascular and renal vascular diseases occurrence, particularly obesity and obesity-related cardiac metabolic risk factors such as glucose and lipid metabolism disorders. Hormone replacement therapy and new oral hypoglycemic drugs contribute to metabolic regulation, which requires sex-specific management. Identifying the key factors and treatment strategies that cause metabolic disorders is critical for strengthening metabolic diseases, cardiovascular and renal complications primary and secondary prevention. Therefore, we should conduct further study from the perspective of sex differences.

Estrogen therapy participated in regulating glucose homeostasis and improving insulin resistance. Determining estrogen tissue specific effects and ER targets will be conducive to the new selective ligand targeting drugs development, thus preventing the type 2 diabetes, metabolic syndrome, obesity occurrence. Study has found that early estrogen replacement therapy can inhibit mitochondrial hydrogen peroxide levels after oophorectomy in rats, prevent oxidative damage to lipids and proteins, increase glutathione peroxidase and catalase activities, and promote brain glucose uptake, thereby protecting against oxidative stress and metabolic disorders caused by oophorectomy.^165^ Estrogen replacement therapy significantly improved insulin sensitivity and decreased diabetes prevalence.^166,167^ Inada et al. used orchiectomy (ORX), E2, or both to treat hyperglycemic male induced early inhibitory factor (ICER) - transgenic (Tg) mice. Study found that with or without ORX, E2 treatment in the early stage of diabetic nephropathy can cause a rapid decrease in blood glucose, a sharp increase in the number of β cells, a decrease in glomerular sclerosis, type IV collagen deposition, and proteinuria, thus alleviating diabetic nephropathy occurrence. E2 treatment was more effective than ORX alone in slowing diabetic nephropathy progression. Although pancreatic islet transplantation can improve glucose levels and eliminate proteinuria in ICER-Tg mice. However, E2 combined with ORX treatment had a greater effect on improving glomerulosclerotic lesions and renal function than pancreatic islet transplantation. As a result, E2 treatment could be a novel therapeutic strategy for improving hyperglycemia and preventing diabetes nephropathy.^168^ Anastrozole, an aromatase inhibitor (Da), protected against kidney damage in male streptozotocin-induced diabetes rats by inhibiting estradiol synthesis, urinary protein, glomerulosclerosis, and tubulointerstitial fibrosis.^169^ More and more evidence suggests that testosterone therapy can help to improve visceral obesity and metabolic syndrome.^170,171^ Testosterone therapy decreased visceral fat mass, plasma insulin and leptin levels, improved glucose and lipid homeostasis, which reduced the cardiovascular diseases related to metabolic syndrome risk in men.^172,173^

Androgen deprivation therapy (ADT) is a first-line treatment and basic management method for advanced prostate cancer patients, which inhibited the androgen/AR signaling function. However, long-term ADT treatment may significantly increase the amount of fat and circulating insulin levels.^174^ In prostate cancer (PCa) patients due to severe testosterone deficiency, resulting in significant insulin resistance and hyperglycemia, as well as increased diabetes and metabolic syndrome risk.^175–177^ Therefore, it is suggested that PCa patients receiving ADT treatment make lifestyle changes in order to prevent insulin resistance and metabolic syndrome. Furthermore, insulin sensitizers combined with anti-androgen drugs could be a more effective method for treating metabolic syndrome and advanced PCa patients.^178,179^ In the future, a new generation of treatment options targeting androgen synthesis or directly targeting AR in combination with ADT will have the potential to better manage PCa patients’ metabolic and cardiovascular risk factors and complications.

The clinical application of both traditional and novel oral hypoglycemic drugs has a wide impact on regulating glucose and cardiovascular metabolic homeostasis. Clinical trials show that metformin can slow the progression of diabetes in pregnant women by 40%.^180^ Raparelli et al. demonstrated that women with type 2 diabetes benefited more from metformin treatment than men in terms of cardiovascular protection. Women treated with glucagon-like peptide 1 (GLP-1) receptor agonists had a lower rate of cardiovascular events than men.^181^ Clinical trials showed that the combination of exenatide and metformin can improve insulin sensitivity, lower insulin resistance, and blood lipid levels, and increase adiponectin levels, which was more beneficial for female patients with type 2 diabetes than male. Therefore, the combination therapy of exenatide and metformin may be more suitable for female patients.^182^ Currently, sodium glucose co-transporter 2 (SGLT2) inhibitor is now more commonly used in men. Study suggests that SGLT2 inhibitor may have a more effective therapeutic response to improve glucose metabolism in men. SGLT2 inhibitor treatment can significantly decrease the atherosclerotic cardiovascular disease, chronic kidney disease and heart failure risk for type 2 diabetes patients, as well as make a protective impact on heart and kidney function.^183,184^ SGLT2 inhibitor treatment did not show sex differences in major cardiovascular adverse events.^185^ Clinical studies revealed that empagliflozin had no significant difference in decreasing the cardiovascular death risk, hospitalization for heart failure, or enhancing cardiovascular prognosis in male and female heart failure patients.^186^ In addition, DELEVER clinical trials have also found that dapagliflozin treatment had similar health benefits in preventing the heart failure deterioration or cardiovascular death in male and female patients with heart failure with mildly reduced or preserved ejection fraction.^187^

In summary, estrogen and androgen therapy, as well as novel drugs (GLP-1 receptor agonists, SGLT2 inhibitors) clinical application have a profound impact on regulating glucose homeostasis and improving metabolic abnormalities. Female patients with type 2 diabetes are more likely to develop cardiovascular diseases. Therefore, clinicians could need pay close attention to related risk factors in order to decrease cardiovascular and metabolic diseases incidence. Sex differences could need be taken into account when selecting treatments for metabolic diseases such as anti-diabetes to guide future basic and clinical studies. Further understanding of sex differences in metabolic disease treatment can help us better target the prevention of metabolic disorders.

## Sex difference in cancer

### Introduction

In recent years, epidemiological studies have emphasized that sex is a noticeable factor that caused differences in the many cancers incidence rate and survival rates, including colorectal cancer, breast cancer, gastric cancer, pancreatic cancer, lung cancer and liver cancer. Studies confirmed that women had a lower incidence and mortality rate for hepatocellular carcinoma compared with men.^188–190^ Gastric cancer is the third leading cause of cancer-related deaths among men and women worldwide. The global male gastric cancer incidence rate was twice that of female, and female gastric cancer treatment prognosis was better.^3,191^ Study data showed that the prevalence of esophageal cancer, hepatocellular cancer, colorectal cancer and pancreatic cancer in men was 4.39 times, 2.89 times, 1.31 times and 1.30 times higher than that in women, respectively.^192^ However, clinical study demonstrated that breast cancer and thyroid cancer were two exceptions, with women having a higher incidence rate than men.^193^

### Mechanism

**Fig. 3:**
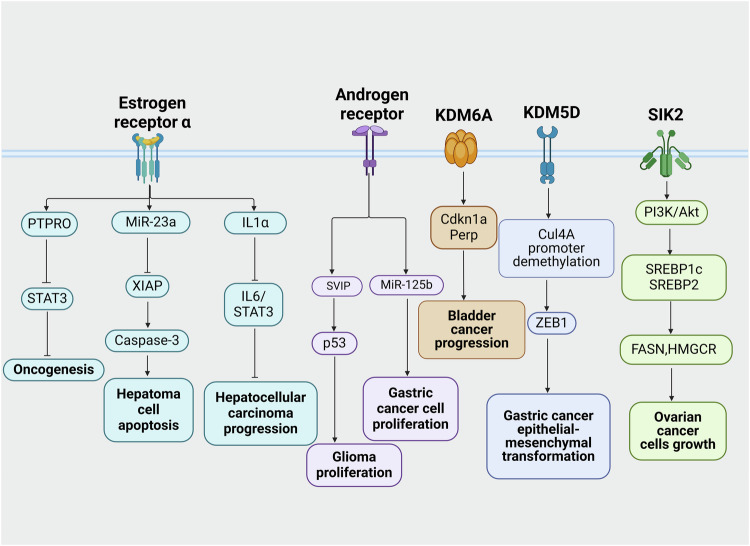
This figure shows the mechanisms of sex differences that regulate cancer, including estrogen, androgen signaling pathways and gene expression. Estrogen receptors affect the occurrence of various tumors such as hepatocellular carcinoma by regulating PTPRO, miR-23a and IL1a. Androgen receptors promote the proliferation of tumor cells by regulating SVIP and miR-125b. KDM6A, KDM5D and SIK2 regulate the expression of many genes and thus affect the progression of many cancers. This figure was created with the aid of BioRender (https://biorender.com/). Akt protein kinase B, Cdkn1a cyclin-dependent kinase inhibitor 1 A, CUL4A cullin 4A, FASN fatty acid synthase, HMGCR hydroxy-3-methylglutaryl-CoA reductase, IL Interleukin, KDM6A X-linked lysine demethylase 6A, KDM5D histone lysine specific demethylase 5D, PERP p53 apoptosis effector related to PMP22, PI3K Phosphoinositide 3-kinase, PTPRO: Protein tyrosine phosphatase receptor type O, SIK2 Salt inducible kinase 2, SREBP1c sterol regulatory element binding protein 1c, SREBP2 sterol regulatory element binding protein 2, STAT3 Signal transduction and activator of transcription 3, SVIP small VCP/p97 interacting protein, XIAP X-linked inhibitor of apoptosis protein, ZEB1 Zinc Finger E-Box Binding Homeobox 1

Male and female cancer incidence and mortality rates may differ primarily include the following mechanisms: traditional sex hormone regulation, gene variations, and epigenetic influences. (Fig. 3).

#### Hormone regulation

In terms of sex hormone regulation, sex hormone receptors activated downstream regulatory genes by binding to ligands to participate in cancer-related signaling pathways. Estrogen signaling pathway played a protective role in the tumors occurrence and development such as liver cancer and colorectal cancer. Multiple studies have supported that the estrogen signaling pathway may have anti-liver cancer effects through regulating liver cancer cells proliferation, apoptosis, and oxidative stress response.^193^ ERα can inhibit human hepatocellular carcinoma cell proliferation and invasion by downregulating MTA 1 transcription.^194^ E2 was activated by ER α and combined with miR-23a regulatory region to induce its expression, thereby downregulating its target gene X-linked inhibitor of apoptosis protein (XIAP), activating caspase-3 activity, and leading to liver cancer cells apoptosis.^195^ E2 antagonized the malignant development of hepatocellular carcinoma cells by activating caspase 1 dependent cell apoptosis, inhibiting inflammatory factors secretion, cell proliferation, and autophagy.^196^ Estradiol decreased liver cell cycle regulators expression and cyclin E kinase activity while activating p53 and p21 expression levels, thus inhibiting liver cancer process.^197^ ERα up-regulated protein tyrosine phosphatase receptor type O (PTPRO) expression, reduced signal transduction dependent on Janus kinase (JAK) and phosphoinositide 3-kinase (PI3K) dephosphorylation, and STAT3 transcriptional activity, thus delaying tumor progression.^198^ In addition, estrogen regulated oxidative stress and inflammatory cells and inflammatory factors expression, thus inhibiting cancer cells development. Estrogen regulated Foxo3a or downregulated ERα expression, activated the Akt/Foxo3a signaling axis, inducing oxidative stress and apoptosis in hepatocellular carcinoma cells. Estrogen can prevent the tumor microenvironment formation by regulating the polarization of macrophages, NK cells, and Th1 cells, as well as the production of inflammatory cytokines TNFα, interleukin-4, and interleukin-6.^199,200^ Estrogen signaling pathway activated ERα promoter, binded to interleukin-1α, regulated interleukin-1α expression through STAT3 and NF-κB signaling, and ultimately inhibited hepatocellular carcinoma progression.^201^ Estrogen can also have a certain protective effect in preventing colorectal cancer by promoting cancer cell apoptosis, inhibiting cancer cell proliferation and metastasis.^202^ Study revealed that male mice colorectal cancer incidence rate was significantly higher than that in female mice. Estradiol inhibited inflammation and colorectal cancer in male mice by activating nuclear factor erythroid 2-related factor 2 (Nrf2)-related pathways.^203^ Estradiol can prevent the loss of SOD and glutathione peroxidase activity as well as inhibit lipid peroxidation, thereby reducing the AP-1 and NF-B activation, lipid peroxide levels, and tumor occurrence.^204–207^ Furthermore, estrogen also promoted cell apoptosis in renal cell carcinoma, whereas androgens and AR increased angiogenesis, proliferation, and invasion of renal cell carcinoma, caused immune cell depletion and dysfunction, thus resulting in the renal cell carcinoma development.^208–215^ Estrogen regulated gene expression, inflammatory factors, and immune cell responses to prevent many cancers progression. Premenopausal women had higher estrogen levels compare to men, which could be the reason for the lower female cancer rates. The protective effect of estrogen on tumor cells needs to be further explored in clinical studies in the future.

Androgens bind to AR and activated the PI3K/Akt pathway, which regulated the cyclin-D and epidermal growth factor receptor (EGFR) signaling pathways, promoting cancer progression.^216^ Study indicated that male glioblastoma patients had a higher incidence rate and a worse prognosis than female patients, which could be due to men having significantly higher androgen concentrations than women, and the activation of androgen receptors in glioblastoma cells promoted cell apoptosis imbalance.^217^ Yu et al. discovered that the AR was widely expressed in glioblastoma. The active AR ligand 5α-dihydrotestosterone (DHT) may contribute to glioblastoma occurrence by reducing the effect of TGFβ receptor signaling on glioblastoma cells growth and apoptosis.^218^ Study has found that AR upregulation promoted glioblastoma proliferation and progression by decreasing small VCP/p97 interacting protein (SVIP) and p53 expression.^219^ Androgens and AR were linked to gastric cancer development. The high incidence of male gastric cancer may be due to AR signaling pathway regulation. Androgens activate the AR/miR-125b signaling pathway in gastric cancer, thereby inhibiting cell apoptosis and promoting cell proliferation.^220^ AR antagonist bicalutamide, can weaken the AR/miR-125b-axis to induce cancer cell apoptosis and inhibit cell growth. Currently, AR targeting strategies have been extensively explored in clinical trials for hepatocellular carcinoma, breast cancer, bladder cancer, and ovarian cancer.^221–225^ In summary, at present, androgens have been shown to promote various cancers development. Therefore, developing therapeutic drugs targeting AR can serve as a new direction for preventing cancer progression.

#### Gene and epigenetic modification

The most obvious genetic difference between sexes is the different copies of the sex chromosomes (X and Y chromosomes). Males and females have significantly different X and Y chromosome gene numbers and mutation rates. A subset of X chromosome genes could prevent X inactivation and protect women from complete function loss due to a single mutation. Andrew et al. found six chrX genes in the non-pseudoautosomal region (PAR): ATRX, CNKSR2, DDX3X, KDM5C, X-linked lysine demethylase 6 A (KDM6A), and MAGEC3, which increased the risk of functional loss mutations in males. It was also discovered that the dual alleles of the EXITS gene in women were beneficial in protecting women from developing tumors risk.^226^

P53 was regarded as the most famous tumor suppressor gene on the autosome. In the p53 compound mutation mouse model, males were more susceptible to invasive cancer and had a lower survival rate than females. Consistent with this, TP53 mutations had a higher incidence in male cancers compared to females, resulting in poorer survival and prognosis in male lung adenocarcinoma. Multiple X-encoded alleles have become regulatory factors for p53 function.^227,228^ KDM6A, DDX3X, and UBA1 were more highly expressed in healthy female tissues than in males. The X chromosome had tumor inhibition function by regulating p53 and mutant alleles expression. The components of the X chromosome p53 pathway network were obvious in unexpressed mutant X alleles. Compared to males, the mutated X allele overall expression was inhibited in female cancers, leading to a higher p53 mutations risk in male cancers.^227,228^ Furthermore, the loss of Y chromosome in male cells has been connected with some cancers development, including lung cancer, glioblastoma and renal tumors.^229–231^ Mutated p53 caused a decrease in mitochondrial number and ATP production, as well as promoted oxidative stress and sustained DNA damage, thereby accelerating cancer occurrence. However, wild-type p53 can inhibit mitochondrial DNA mutations and promote mitochondrial biogenesis.^232,233^ Mitochondria may be more adapted to female environments, an increase in ROS further promoted p53 accumulation.^234^ Therefore, p53 mutations and sex chromosomes are regarded as important factors in molecular pathways that gave rise to sex differences in cancer risk. Y chromosome gene expression (EDY) is a distinct feature that can easily cause cancer in men. Studies have discovered that Y chromosome loss was a common early event in urothelial bladder cancer.^235^ Smoking induced Y chromosome loss. The loss of the Y chromosome in blood cells was related to increased non-hematological tumors risk. South Korea clinical study found that the programmed cell death ligand 1 (PD-L1) expression was negatively correlated with male proximal colorectal cancer patients, while female proximal colorectal cancer were significantly associated with high expression of dMMR/MSI and EGFR.^236^ Therefore, this could be a potential mechanism for the disparity in colorectal cancer incidence and mortality rates among men and women.^237^ In a word, these studies emphasized the critical and distinct effect of sex chromosomes in tumor suppression, while also providing new insights into the potential mechanisms of sex differences in cancer.

Molecular indicators of sex differences in glioblastoma were identified in the Cancer Genome Atlas (TCGA) and the Chinese Glioma Genome Atlas (CGGA) databases. The analysis revealed that autosomal genes NOX, FRG1BP, and AL354714.2, as well as X-linked genes PUDP, KDM6A, DDX 3X, and SYAP 1, had different DNA methylation and expression profiles in male and female glioblastomas.^238^ KDM6A is a bisexual dimorphic gene. Wild-type KDM6A can inhibit tumor cell activity. KDM6A knockout mice decreased p53 target genes Cdkn1a and Perp expression, increasing female mice bladder cancer risk. Study has revealed that the effect of the X chromosome was mainly due to the XCI escape gene KDM6A, which reduced female bladder cancer risk through the epigenetic mechanism of regulating the p53 signal pathway. Developing treatments that target the KDM6A-dependent epigenome could narrow the difference in male and female bladder cancer risk by decreasing male bladder cancer risk.^239^ EGFR is closely related to DNA methylation and ovarian cancer development. Samudio Ruiz et al. found that the presence of a large amount of epidermal growth factor receptor (EGFR) activators in the ascites of ovarian cancer leaded to sustained overexpression of EGFR and its ligands in the tumor microenvironment. Long term exposure to EGF or sustained activation of EGFR may increase DNMT activity and global DNA methylation. DNMT inhibitors/low methylation agents 5-aza-2 ‘- deoxycytidine (AZA) and EGFR targeted drugs prevented ovarian cancer progression by altering EGF mediated DNMT activity and overall methylation.^240–243^ Histone lysine specific demethylase 5D (KDM5D) could be one of the important epigenetic modifiers that gave rise to male gastric cancer poor prognosis. Study has found that KDM5D downregulation in gastric cancer cells inhibited cullin 4A (Cul4A) promoter demethylation, resulting in an increase in Zinc Finger E-Box Binding Homeobox 1 (ZEB1) and mesenchymal marker expression, a decrease in p21 and p53 expression, thereby promoting male gastric cancer epithelial-mesenchymal transition. Therefore, KDM5D is expected to become a promising male gastric cancer therapeutic target.^244^ H3K27me3 demethylase ubiquitously transcribed X (UTX) is a sex specific tumor suppressor in t-cell acute lymphoblastic leukemia (T-all). H3K27me3 demethylase UTX undergoes repeated mutations in male T-all. However, UTX escaped and became inactive in female T-all mother cells and normal T cells. This may be the reason why men had a higher risk of developing T-all than women. Study has shown that H3K27me3 demethylase UTX, as a tumor suppressor gene, made a protective effect on T-all.^245^ OTUB1 (ovarian tumor associated proteinase B1) is an OTU superfamily deubiquitination enzyme that specifically inhibited the deubiquitination of c-MYC proteins linked to K48 and K63, thereby accelerating cardiomyocyte apoptosis, inflammation response, and oxidative stress. Study has discovered that knocking down the deubiquitination enzyme OTUB1 can alleviate myocardial fibrosis and myocardial atrophy by inhibiting doxorubicin (DOX) in the treatment of ovarian cancer-induced cardiomyocyte apoptosis, inflammation, and oxidative stress, as well as prevent DOX-induced cardiovascular dysfunction in mice. It has shown that OTUB1 could be a promising therapeutic target for protecting against DOX-induced cardiac toxicity in ovarian cancer mice. However, in DOX-induced heart failure, whether OTUB1 can inhibit cardiotoxicity by regulating gene expression in other cell types needs further experimental verification.^246^ Salt inducible kinase 2 (SIK2) is a tumor-promoting factor in ovarian cancer. In vitro and in vivo experiments have revealed that SIK2 upregulated the sterol regulatory element binding protein 1c (SREBP1c) and sterol regulatory element binding protein 2 (SREBP2) expression via the PI3K/Akt signaling pathway, promoting major lipogenic enzyme fatty acid synthase (FASN) and cholesterol synthase hydroxy-3-methylglutaryl-CoA reductase (HMGCR) transcription, thereby enhancing fatty acid and cholesterol synthesis and contributing to ovarian cancer cell growth. SIK2 overexpression promoted ovarian cancer cells intraperitoneal metastasis. SIK2 activation also increased AMPK-induced acetyl Coa carboxylase phosphorylation, which accelerated ovarian cancer progression. Therefore, SIK2 would be regard as a potential target for ovarian cancer diagnosis and treatment.^247–249^

For a long time, miRNA has been important in regulating female liver cancer via the estrogen and receptor pathway. Li et al. found that ERα protein level was decreased in female hepatocellular carcinoma. This may be due to the fact that the elevation of p53 increased miR-18a level, which inhibited ERα protein expression and thus accelerated female liver cancer occurrence.^250^ Furthermore, it was discovered that miRNA-206 expression was elevated in ER negative tumors and miRNA-206 down-regulated ERα expression by targeting it. Overexpression of miRNA-221, miRNA-222 and miRNA-206 induced the ERα positive cells proliferation. In turn, ERα directly inhibited miRNA-221 and miRNA-222 by recruiting co-inhibitors NCoR and SMRT in the negative feedback loop. Therefore, these miRNAs may serve as important factors in promoting the transformation of ERα-positive precursors to ERα-negative tumors. The ERα and miRNA-221, miRNA-222 and miRNA-206 molecular pathways and their interactions need to be further explored and validated.^251^ MiR-141 and miR-200 a/b/c were reported to be the most significantly overexpressed miRNAs in ovarian cancer, while miR-199, miR-140, miR-145, and miR-125b were significantly downregulated. As a result, these miRNAs may be useful as diagnostic biomarkers for ovarian cancer.^252^ Tang et al. discovered that miRNA-423-5p expression was decreased in ovarian cancer patients tissue and plasma. Increased ovarian tissue and plasma miR-423-5p expression can slow ovarian cancer progression by inhibiting cell proliferation, colony formation, and invasion. However, the direct target of miR-423-5p in ovarian cancer will need to be further clarified in the future. In addition, the expression level of miR-423-5p in ovarian cancer patients and its correlation with clinical outcomes will need to be further explored.^253^ Long chain non-coding RNA FTX (lnc-FTX) is regarded as a regulatory gene in predicting the risk of sex differences in hepatocellular carcinoma patients. Liu et al. indicated that the expression level of lnc-FTX in female liver was higher than that in male liver, and it was positively correlated with patient survival. However, the expression of lnc-FTX was significantly decreased in female hepatocellular carcinoma. Lnc-FTX inhibited hepatocellular carcinoma growth and metastasis by competitively binding to miR-374a and mini-chromosome maintenance complex component 2 (MCM2), which inhibited Wnt/β-catenin signaling and DNA replication. This finding could provide new insights into the mechanisms that prevent hepatocellular carcinoma in women. However, further studies will be worthy to be explored whether sex hormones are involved in the regulation of lnc-FTX in inhibiting hepatocellular carcinoma progression.^254^ Qu et al. found that circ-ASPH was widely expressed in glioma cells. The upregulation of circ-ASPH regulated AR expression in Glioma by targeting the miR-599/AR/SOCS2-AS1 signaling pathway, thus promoting glioma development. As a result, circ-ASPH may be a promising target for future glioma treatments.^255^ In summary, there are significant differences in the DNA methylation and demethylation expression of tumor-related genes between males and females. The key enzymes of DNA methylation, demethylation and histone modification, as well as non-coding RNAs, exist sex differences in multiple cancers regulation. Further study of epigenetic-related drugs based on the different characteristics of these genes expression in men and women, and then validation in clinical trials, which would have promising value for improving clinical cancer treatment and prognosis.

### Clinical implications

Sex not only affects the incidence rate and mortality of different cancers, but also has obvious differences in the diseases treatment response and effect. Hormone replacement therapy and immunotherapy can also play different roles in tumor prevention due to sex differences. Sex differences could affect tumor drugs pharmacokinetics and their response to local treatments such as radiotherapy. Male and female wild-type mice were found to be less affected by paclitaxel induced neurotoxicity and radiation induced cardiac toxicity compared to male mice treated with paclitaxel.^256^ Female rats have a lower risk of adverse reactions such as vasculitis and pneumonia caused by radiation therapy.^257^ The mechanism may be that RhoB can reduce the sensitivity of female mouse hearts to radiation therapy and increase the radiation toxicity of male mice. Therefore, RhoB has a key value in promoting estrogen dependent cardiac protection in female mice.^256^

Radiation therapy usually used in patients undergoing breast-conserving therapy and regional lymph node involvement patients, which is essential to improve breast cancer survival. However, radiation to the heart from radiotherapy increased breast cancer-related cardiac insufficiency incidence and mortality. Radiation therapy can cause damage to heart tissue in as little as minutes. It is manifested in endothelial cell injury that aroused rapid recruitment of inflammatory cytokines (monocyte chemokines, tumor necrosis factor, and interleukin) and pro-fibrotic cytokines (platelet-derived growth factor, transforming growth factor β) in myocardial tissue, resulting in inflammatory response activation, promoting ROS production and increasing vascular permeability.^258^ In addition to serving as the second messenger signal in cells, ROS stimulated pro-fibrotic proteins, chemokines, cytokines expression in endothelial cells and participate in the MAPK and nuclear factor-κB (NF-κB) signaling pathway regulation.^259^ ROS can also cause mitochondrial calcium overload, cell membrane swelling and apoptosis factors release, thus participating in chronic inflammatory damage.^260^ Radiation therapy can give rise to fibroblasts premature activation after only 3-4 cell division cycles. Meanwhile, the activity of these mitotic cells to produce interstitial collagen I, III, and IV was 25 to 26 times higher than normal.^261^ These factors together increased myocardial fibrosis. Furthermore, radiation increased focal ischemia, oxidative stress, and DNA damage, which further interfered with the cardiomyocytes normal function and gave rise to cardiomyocytes apoptosis.^262,263^ These acute and chronic changes together resulted in cardiac function reduction and accelerated heart failure progression. Anthracyclines exert a critical role in breast cancer chemotherapy. Anthracycline-induced cardiotoxicity can occur through a variety of mechanisms. Increased oxidative stress and reactive oxygen species formation were thought to be the primary causes of heart failure. Anthracyclines can lower the levels of antioxidant enzymes GSH-Px and SOD in the myocardium, so that free radicals and superoxides cannot be removed in time, and thus damage cardiomyocytes. Anthracyclines generated free radicals via non-enzymatic pathways.^264^ Anthracyclines could also specifically bind to the phospholipid cardiolipin and accumulate within the mitochondria, disrupting the membrane and electron transport chains.^265^ Moreover, anthracyclines can interact directly with iron to form reactive iron complexes and contribute to iron cycling between divalent and trivalent, which has been linked to ROS production and iron homeostasis changes.^266^ These changes above accelerated heart failure progression. Trastuzumab prolonged metastatic HER2-positive breast cancer patients overall survival. However, it could also trigger heart failure. It was found that the cardiac dysfunction induced by trastuzumab was reversible, mainly manifested by cardiomyocyte hypertrophy, mild interstitial fibrosis, and focal vacuolar changes.^267^ Pathogenetic mechanisms were mainly caused by mitochondria and contractile proteins structural and functional changes, however, cardiomyocyte death was rare.^268^ Trastuzumab suppressed BCL-XL expression and up-regulated BCL-XS activity. BCLXL inhibited apoptosis, whereas BCL-XS promoted apoptosis. As a result, apoptosis imbalance activated the mitochondrial apoptotic pathway, causing mitochondrial dysfunction and cell death, which led to cardiac insufficiency.^269^ ErbB2 knocked out mice heart showed ventricular dilation and cardiac wall thinning.^270^ Trastuzumab can inhibit the neuregulinin-1/ErbB2 axis to weaken myocardial repair mechanism and lead to myocardial injury.^271^ Furthermore, preclinical data suggests that activating the HER2 signaling pathway reduced oxidative stress in cardiomyocytes. Trastuzumab inhibited the HER2 signaling pathway, thus giving rise to oxidative balance disruption and cardiovascular dysfunction.^272^

In recent years, estrogen replacement therapy has shown promising results in improving the prognosis of cancers such as liver, lung, and kidney cancer. Multiple clinical studies have observed that hormone replacement therapy during menopause reduced female hepatocellular carcinoma patients risk and prolonged their survival. Therefore, exploring drugs targeting the estrogen signaling pathway could have potential clinical significance for the treatment of liver cancer patients.^273,274^

Estrogen is metabolized primarily by the liver. Therefore, its metabolites also have a certain impact on liver cancer cells proliferation. Study has shown that overexpression of the liver-specific cytochrome P450 1A2 (CYP1A2) promoted the conversion of E2 into the metabolite 2-methoxyestradiol (2-ME), decreased the vascular endothelial growth factor (VEGF) and Bcl-2 expression, which contributed to induce cell apoptosis, and alleviate liver cancer cells proliferation and progression.^275^ Sorafenib is the only effective first-line drug approved for the advanced hepatocellular carcinoma treatment, which participated in the anti-proliferation, anti-angiogenic and pro-apoptotic processes of liver cancer cells.^276^ Combining E2 with sorafenib can improve the therapeutic effect in patients with liver cancer cells.^275^ Stabile et al. demonstrated that the combination of aromatase inhibitor anastrozole and nonsteroidal anti-inflammatory drug aspirin could significantly reduce lung tumors when compared to single drug therapy and that the mechanism of action was mainly to decrease lung cancer risk by inhibiting estrogen and COX2 activities, pro-inflammatory cytokines, and macrophage recruitment. It was particularly effective at preventing lung cancer in female ovariectomized mice and women with lung inflammation risk factors.^277^ Furthermore, anti-estrogen therapy or hormone replacement therapy can prevent lung cancer development by blocking ERs, making it particularly effective for lung cancer patients who express ERs.^278,279^ Previous studies and the latest meta-analysis have indicated that female hormone replacement therapy had a protective effect on gastric adenocarcinoma. Hormone replacement therapy can reduce gastric cancer risk by more than 28% compared to patients who did not receive it. Both estrogen therapy alone and estrogen-progesterone therapy decreased gastric cancer risk compared to non-users.^280,281^ In conclusion, it is necessary to carry out more estrogen replacement therapy clinical trials in different cancers male and female patients to find the more suitable population for the treatment, so as to better explore the application value and therapeutic effect of estrogen replacement therapy.

Drug therapy that targets androgens and their receptors has the potential to prevent glioblastoma and prostate cancer. AR antagonists enzalutamide and bicalutamide can not only promote cell death in glioblastoma, but enzalutamide can also alleviate glioblastoma volume and proliferation, thereby inhibiting glioblastoma progression. Therefore, AR antagonists could be regarded as promising drugs for clinical glioblastoma treatment.^282,283^ Orozco et al. discovered that dutasteride, cyproterone, and flutamide can significantly reduce glioblastoma cell proliferation and invasion. Moreover, the combination therapy of Dutamide and Fluticasone is the most effective strategy for inhibiting glioblastoma cell proliferation. Therefore, the study indicated that the synergistic treatment of AR antagonists and 5α-reductase inhibitors could be a more promising glioblastoma therapeutic strategy.^284^ Zalcman et al. demonstrated that afatinib, an EGFR kinase inhibitor, can block AR activation and nuclear translocation in glioblastoma cells. As a result, the combination of AR antagonists and EGFR kinase inhibitors was expected to be a more effective glioblastoma therapeutic method. In addition, natural compounds curcumin, ALZ 003, and Cedrol inhibited glioblastoma cells proliferation by blocking AR signaling, and could be used as new drugs for the future glioblastoma treatment, thus opening up new avenues for glioblastoma prevention.^285–287^ Study found that AR antagonists bicalutamide, cyproterone, enzalutamide, and flutamide participated in prostate cancer clinical treatment.^283,284,288,289^ Seviteronel, a selective CYP 17 lyase inhibitor and androgen receptor inhibitor had significant anti-tumor activity and had completed phase I and II clinical trials for prostate cancer patients.^290,291^

Immune checkpoint blockade therapy, which includes the inhibition of programmed cell death 1 (PD-1) or ligand 1 (PD-L1) and cytotoxic T lymphocyte antigen-4 (CTLA-4), helps to extend various cancer lineages survival. The treatment efficacy of immune checkpoint blockade in tumor patients varies by sex and age.^292^ Two systematic reviews and meta-analyses found that anti-PD-1/PD-L1 monotherapy was more effective in men, whereas the combination of anti-PD-1/PD-L1 chemotherapy was more beneficial to women with advanced lung cancer.^293^

At present, many drugs have important clinical significance in ovarian cancer treatment. Polyethylene glycol liposome doxorubicin (PLD) is a nano-doxorubicin anticancer agent. PLD can significantly reduce conventional doxorubicin’s cardiotoxicity. Because of its significant anti-cancer efficacy and tolerability, the 2018 National Comprehensive Cancer Network Guidelines recommended PLD as a first-line ovarian cancer chemotherapy drug. Clinical trials have also showed the significant clinical value of PLD combined with carboplatin as a first-line refractory recurrent ovarian cancer chemotherapy regimen.^294^ Kelm et al. found that Withaferin A treatment reduced plasma angiotensin II and angiotensin II type 1 receptor-induced inflammatory factors expression in tumor-bearing mice, decreased cardiac fibrosis, and enhanced cardiac function, suggesting that it could play a protective role in cardiac remodeling and dysfunction caused by ovarian cancer xenotransplantation.^295^ PDE10A is expressed in human tumor cell lines. Chen et al. found that PDE10A inhibition not only reduced ovarian cancer cells growth and induced cell apoptosis, but also enhanced the chemotherapy efficacy of doxorubicin (DOX) on ovarian cancer, preventing DOX induced cardiac toxicity and dysfunction. Inhibiting PDE10A alleviated cardiomyocytes DNA damage and mitochondrial dysfunction and improved DOX-induced cardiomyocyte death and cardiac fibrosis. PDE10A inhibition reduced DOX-induced myocardial atrophy in C57Bl/6 J mice by blocking cAMP/PKA (protein kinase A) and cGMP/PKG (protein kinase G) dependent signaling pathways. As a result, inhibiting PDE 10 A might be deemed as a promising anticancer therapeutic strategy.^296^ Raab et al. revealed that a new selective SIK2 small molecule inhibitor, MRIA9, could enhance the sensitivity of ovarian cancer cells and patients to paclitaxel treatment. The study suggests that future development of inhibitors that selectively target SIK2 could contribute to prevent paclitaxel resistance in ovarian cancer patients, improving their prognosis.^297^

Together, these findings highlight that sex factors could need to be taken into account in clinical trials in order to better investigate sex differences in cancer management and treatment. Individualized therapeutic regimens based on sex differences in disease contribute to improve cancer patients’ symptoms and prognosis.

## Sex differences in autoimmune diseases

### Introduction

Autoimmunity means the loss of self-tolerance to the state of immune response and autoantigen. The disease state result from autoimmunity that is called “autoimmune disease”, which often leads to organ and tissue damage. Immune disease is the most common type of disease showing sex dimorphism. sex dimorphism in immune diseases affects not only the incidence of the disease but also in the course and severity of the disease.^123^ Women account for approximately 78% of patients with autoimmune diseases, which is one of the main reasons of morbidity and mortality in senior female.^298–300^ Epidemiological studies have revealed that women were susceptible to have autoimmune diseases like systemic lupus erythematosus, Sjogren’s syndrome, and multiple sclerosis. Women’s susceptibility to autoimmune diseases may be caused by stronger congenital and adaptive immune responses, which is shown in that women have a lower incidence of severe infection and better response to vaccine antibodies than men.^301–303^ As a chronic autoimmune disease, multiple sclerosis has been the culprit of neurological diseases in the younger, whose typical feature is the demyelinating lesions of the central nervous system.^304^ Multiple sclerosis is caused by an absence of self-tolerance to myelin and central nervous system antigens, which typically results in the continuous activation of autoreactive T lymphocytes.^305^ Furthermore, the pathological mechanism of multiple sclerosis disease is characterized as the disrupt communication between T cells, B cells, effectors, and regulatory subsets.^306^ Multiple sclerosis has a sex-related susceptibility, with women having a higher incidence than men, by a ratio of 2-3:1.^307^ In addition, a comparison of consecutive cross-sectional studies revealed that the proportion of women to men in multiple sclerosis cases increased gradually over time.^308^ Systemic lupus erythematosus is featured as abnormal immune activity caused by gene, epigenetic, and hormonal factors, but the exact pathogenesis is unknown.^309^ Systemic lupus erythematosus is more common in women, with a male-to-female ratio of 8:1 to 15:1.^310^ The prevalence of systemic lupus erythematosus increased dramatically during the childbearing, but decreased significantly before puberty and after menopause.^311^ Sjogren’s syndrome is featured as lymphocyte infiltration of the exocrine glands. T and B cells were the most common infiltrating cells.^312,313^ Overactivation of B cells is regarded as the primary cause of primary Sjögren’s syndrome.^313^ Multiple studies suggested that effector T cells, particularly Th1, Th17, and follicular helper T cells (Tfh) participated in the primary Sjögren’s syndrome development.^312^ Most of patients with systemic lupus erythematosus are between 40 and 50 years old, with a prevalence rate of 0.29%-0.77%. The elderly have a prevalence rate of 3%-4%, and 9 times higher in women than in man.^314^

### Mechanism

**Fig. 4:**
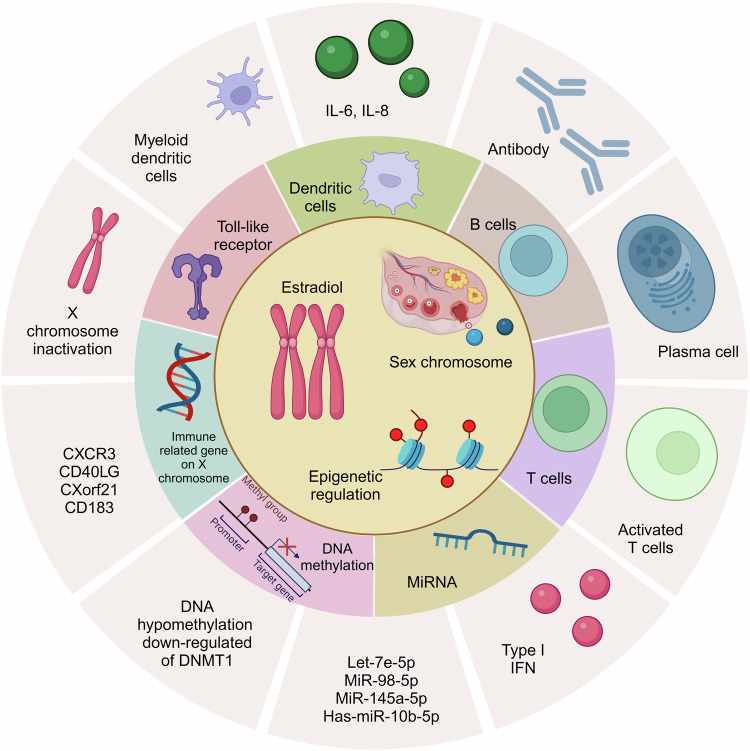
The pathogenesis of sex-based autoimmune diseases. Estrogen, sex chromosomes and epigenetic regulation are the main causes of sex-related autoimmune disease. These factors can play a role by affecting B cells, T cells, dendritic cells, Toll-like receptors, related immune genes on X chromosome, DNA methylation and miRNA. Affecting B cells can make B cells mature and increase the secretion of plasma cells and antibodies. Estrogen also affects the activation of DC through TLR signal pathway. These factors can lead to the increase of inflammatory factors IL-6, IL-8 and type I interferon resulting in autoimmune activation. Related immune genes encoded by X chromosome, such as CXCR3, CD40LG, CXorf21, CD183, also play an important role in immune activation. In addition, due to the increase of X chromosome inactivation escape genes, the number of X chromosome may be an important reason for sex bias immune function. In epigenetic regulation, estrogen can lead to global DNA hypomethylation by down-regulating the expression of mRNA and protein in DNMT1. Therefore, the decrease of DNA methylation at X promoter site will lead to abnormal expression of X-linked genes. In addition, miRNA can play a role in autoimmune diseases in a sex-specific manner. Let-7e-5p, miR-98-5p, miR-145a-5p, has-miR-10b-5p can exert immune response by regulating the expression of inflammation-related genes such as IL-1 and IFNα. This figure was created with the aid of BioRender (https://biorender.com/). CD40LG Chemokine receptor 40 Ligand, CD183 Chemokine receptor 183, CXCR3 C-X-C motif chemokine receptor 3, CXorf21 Chromosome X open reading frame 21, DC Dendritic cells, DNMT DNA methyltransferase, IFN interferon, IL interleukin, TLR Toll-like receptor

Autoimmune diseases, as one of the most common diseases showing sex dimorphism, need to be explored the mechanism of sex differences in autoimmune diseases. Currently, sex hormones, sex chromosomes, and epigenetic mechanisms play vital roles in sex-related autoimmune diseases.^123,315–318^ (Fig. 4)

#### Hormone regulation

The occurrence and function of congenital and adaptive immune responses is influenced by sex hormones., so the imbalance of sex hormones can lead to immune response disorders and autoimmune diseases.^319^ Given the potential mechanism of immune response changes in women’s preferences, estrogen is widely assumed to be the cause of autoimmune diseases. Thus, we concentrate on the function of estrogen in autoimmune diseases associated with sex. Mouse macrophages exposed to low levels of estradiol for 16 hours increased LPS-induced TNFA, interleukin-1, and interleukin-6 gene expression, thus enhancing the proinflammatory ability of macrophages and monocytes.^320^ Similarly, low estradiol levels boost immunological response and interleukin-1 activity in male monocytes.^321^ It has been demonstrated that estrogen promoted B cells maturation and stimulated antibodies secretion, increasing the number of bone marrow progenitor B cells and the survival rate of spleen B cells, thus speeding up the onset of autoimmune responses.^322^ Furthermore, reduced estrogen levels can enhance type 1 helper T cells (TH1) immune response, whereas high levels of estrogen can boost type 2 helper T cell (TH2) cell and humoral immunity. Although autoreactive T and B lymphocytes are the focus of autoimmune diseases, numerous studies have shown that innate immune cells are crucial in the autoimmune diseases development.^323^ Dendritic cells (DC), especially plasma cell-like DC (pDC), are activated in autoimmune diseases. In systemic lupus erythematosus, autoantigens produced type I interferon (IFN) through the Toll-like receptor (TLR)-7 or TLR-9 pathway, which triggered autoimmune response.^324^ Hormone receptor gene expression analysis showed that the peripheral blood mononuclear cells of patients suffering from systemic lupus erythematosus exhibited an increase in the expression of ERα mRNA, while ERβ expression decreased. Studies have shown that estrogen had distinct effects on the pathophysiology of systemic lupus erythematosus due to it regulated different ERs.^325^ ERα depletion weakened glomerulonephritis and anti-double stranded DNA (dsDNA) antibodies development and extended NZB × NZWF1 mice survival, while ERβ deficiency did not affect lupus pathological feature.^326,327^ Activated ERβ was found to bind directly to interleukin-17A and interleukin-21 genes promoters, upregulating interleukin-17 and interleukin-21 transcription levels and helper T cells (Th) 17 expression, which may lead to an increased autoimmune thyroiditis mice immune response.^328^ However, ERα signal transduction pathway had beneficial anti-inflammatory effects in arthritis and multiple sclerosis mouse models.^328,329^

NKanda et al. showed that estradiol enhanced anti-dsDNA antibodies and IgG production in active systemic lupus erythematosus patients, accelerating systemic lupus erythematosus progression.^330^ In addition, study found that BALB/c lupus mice given E2 expressed the anti-DNA antibody transgene encodes H chain and gave rise to increased Bcl-2 expression, thereby improving autoimmune reactive B cells survival.^331,332^ E2 treatment promoted autoimmune reactive B cells survival and autoantibodies production by increasing B cell activating factor level in immune cells, thus promoting systemic lupus erythematosus pathogenesis.^333^ Studies have shown that calcineurin, a marker of T cell activation through ER, was expressed more strongly in T cells of female systemic lupus erythematosus patients treated with E2 compared to male. Similarly, ERα and ERβ agonists could also increase calcineurin and CD154 level in systemic lupus erythematosus patients’ T cells, resulting in T cell activation and enhancement of autoimmune response.^334,335^ Furthermore, E2 can stimulate T cells to express CD40 ligand in systemic lupus erythematosus patients, indicating that estrogen-dependent increase of CD40L expression can overstimulate systemic lupus erythematosus T cells and participate in the systemic lupus erythematosus occurrence.^336^ E2 treatment increased autoantibodies and TH2 cytokines levels in wild-type mice, which induced lupus phenotype, accelerated kidney damage and death.^337^ Several DC subsets expressed different ER patterns and affected ERα signal transduction.^324^ MHC-II and pDC-TREM regulated the type I IFN production and accelerated autoimmune response. E2 treatment enhanced the differentiation of MHC II and CD86 DC on cell surface, and increased interleukin-12, interleukin-18 and IFN-γ levels, thus promoting innate immune response.^338^ However, the differentiation of pDC and the endogenous expression of MHC-II and pDC-TREM decreased in ERα-deficient lupus susceptible mice, which inhibited the autoimmune reaction of ERα-deficient lupus susceptible mice.^339^ E2 enhanced TLR-7 and TLR-9 dependent production of IFN-α in postmenopausal women pDC, thereby modulating pDCs innate function.^340^ Cunningham et al found that TLR-9 increased the expression of interleukin-6 and MCP-1 produced by DC in wild-type mice, however, decreased in ERα-KO mice. Study has indicated that ERα was related to TLR signal transduction. ERα deficiency can reduce the inflammatory response stimulated by TLR9, which in turn had a protective effect against autoimmune disease.^341^ IFN-α had a variety of immunomodulatory effects and could exert noticeable impacts on systemic lupus erythematosus progress. Compared with healthy men, TLR-7-mediated IFN-α increased in peripheral blood lymphocytes of healthy women, resulting in an increased incidence of female systemic lupus erythematosus. Furthermore, TLR-7-mediated IFN-α secretion was enhanced by the delivery of recombinant IFN regulatory factor 5 (IRF5) protein into human pDC, whereas IRF5 mRNA expression and IFN-α production were reduced in pDC through ER gene knockout, resulting in a decreased autoimmune response.^342^ Furthermore, studies have confirmed that estrogen increased STAT1 expression, thus inducing IFN-stimulated gene expression and up-regulating TLR-8 level, resulting in enhanced immune response.^343^ In summary, these data show that estrogen affects DC activation and IFN production through TLR signal pathway, thereby activating the immune response and contributing to autoimmune diseases occurrence. There are significant differences in adult women estrogen levels during puberty, pregnancy and menopause, but there is still a lack of relevant research and evidence. In the future, it is imperative to further investigate the effect of estrogen in sex-related autoimmune diseases during the puberty, pregnancy and menopause period. In addition, we suggest that human innate immune cells and sex hormones need to be comprehensively studied to understand accurately the role of specific hormones on their transcriptome, methylation group, chromatin landscape and immune function.

#### Sex chromosome

The difference in estrogen level can only to some extent explain the sex dimorphism of immune diseases because even in childhood or postmenopausal women with no difference in estrogen levels between sexes, sex bias in women with autoimmune diseases is often observed.^344^ These variations could be buried in sex chromosomes, which participated in different autoimmune response.^345,346^ The mouse model known as “tetranuclear genotype” (FCG) can be applied to isolate sex chromosomes and phenotypic effects induced by gonadal hormones.^347^ This separation eliminates the interference of sex hormone levels to study the effect of sex chromosomes on sex disparities in the disease. By comparing ovariectomized XX and XY− mice with castrated XXSry and XY−Sry mice, it was found that mice with XX sex chromosomes had increased double-stranded DNA autoantibody levels and immune cell activation, leading to increased susceptibility and severity of pristane-induced lupus in mice.^346,348^

Klinefelter syndrome (XXY) has a 14-fold higher risk of systemic lupus erythematosus or Sjögren’s syndrome than euploid women (XX), and Turner syndrome (X, O) has a lower systemic lupus erythematosus incidence than Klinefelter syndrome (XXY) patients.^349–351^ The X chromosome encoded 1100 annotated genes, the majority of which were unrelated to sex-related genes, whereas the Y chromosome contains a substantial amount of significant sex-specific genes involved in germ cell differentiation and masculinization.^352,353^ Uneven gene doses on chromosomes have different roles in regulating immune homeostasis and tolerance. Most of X chromosomes genes were related to the development of immune cells, congenital and adaptive immune responses.^354^ To sum up, the female X chromosome rich in immune-related genes may raise female autoimmunity susceptibility, especially systemic lupus erythematosus and Sjögren’s syndrome. In addition, there is obvious genetic inequality between females (XX) and males (XY). The X chromosome has three times the total DNA of the Y chromosome. The number of transcriptionally active genes in the X chromosome is more than 100 times that of the Y chromosome. To alleviate these inequalities, epigenetic silencing was randomly performed on X chromosomes from men or women through X chromosome inactivation (XCI) process in the early phase of embryogenesis.^355,356^ The assessment of human sex gene expression shows that about 20% of X-resident genes escape inactivation, leading to rich expression of active proteins in women and creating opportunities for autoimmune activation. Therefore, the increase in XCI escape genes may cause sex differences in immune function.^357,358^ To summarize, these findings support the link between sex chromosomes and autoimmune diseases development. It is not clear how the maintenance mechanism of XCI varies according to immune cells type and how they change with age. In addition, it is unclear how many X-inactivation-specific transcriptional interacting proteins function to maintain XCI in immune cells and, in turn, how they promote abnormal escape in specific women’s preferred immunity. The solution of these problems is very important for us to understand the sex differences in immune diseases in precision medicine, and will ultimately provide key mechanism perceptions of the causes of women’s susceptibility to autoimmune diseases.

#### Gene regulation

It has been demonstrated that immune genes on the X chromosome can avoid silence and participate in autoimmune diseases pathogenesis. CD40LG (CD154), encoded by the X chromosome, is primarily a type II transmembrane protein found on T helper cells. It bound to CD40, which was expressed in antigen-presenting cells and activated DC and B cells, causing pro-inflammatory responses. In DC, CD40LG helped to up-regulate additional stimulatory molecules level and promoted antigen cross-presentation, which drove CD4 and CD8T cell responses. In B cells, CD40LG was involved in driving the class switch of immunoglobulins, germinal center formation, and plasma cells activation and production.^359^ Compared with the healthy donor, CD40L was up-regulated in salivary and lacrimal glands of patients with Sjögren’s syndrome, and enhanced adhesion molecule-intercellular adhesion molecule-1/CD54 expression, thus promoting autoimmune diseases occurrence.^360^ Meanwhile, CD40LG exhibited variable XCI escape in primary CD3T cells stimulated in vitro from healthy female donors and systemic lupus erythematosus patients, as well as abnormal overexpression in primary T cells from systemic lupus erythematosus and Sjögren’s syndrome women, indicating that CD40LG encoded by the X chromosome was linked to autoimmune diseases development.^361–363^ CD40L also participated in the pathogenesis of Sjogren’s syndrome, and the fact that many female T cells express biallelic CD40L may explain sex differences.^362^ Overexpression of CD40 leaded to high autoantibody titer and enhanced autoimmune response.^364,365^ Taken together, these findings highlighted the link between CD40LG escape, immune activation, and autoimmune diseases.^366^

Toll-like receptor (TLR) is a type I transmembrane protein that detects the molecular pattern of microbial or cell damage fragments, thereby triggering immune response. Abnormal signal transduction of TLR7 promoted systemic lupus erythematosus and Sjögren’s syndrome pathophysiological processes. The increase in IFN-α expression caused by the TLR7 signal resulted in systemic lupus erythematosus occurrence, which was positively associated with disease activity.^367^ Furthermore, TLR7 mediated B cells activation and overexpression to promote autoantibodies level, which is closely related to systemic lupus erythematosus pathogenesis.^368^ The expression of TLR7 and inflammatory markers in salivary glands was markedly increased in primary Sjögren’s syndrome patients and positively associated with the expression of TNF, LT-a, CXCL13 and CXCR5.^369^ TLR7 mainly induced pDC to produce type I interferon and activated autoimmune reaction through interferon regulatory factor 7 (IRF7) transcription factors.^370,371^ TLR8 is another X-linked endonucleic acid receptor, which was abnormally increased in immune cells of female Sjogren’s syndrome, accompanied by activation of type 1 IFN response, promoting inflammation and exacerbating Sjogren’s syndrome occurrence.^372^ Males had faster systemic lupus erythematosus disease progression and higher TLR7 and TLR8 expression as a result of TLR7 and TLR8 translocation from the X to Y chromosomes. The moderate increase of TLR7 promoted autoreactive lymphocytes and myelocytes proliferation, however, excess TLR7 increased acute inflammatory responses and the number of inflammatory associated dendritic cells, leading to autoimmune response activation.^373–375^ Similarly, compared with XY immune cells, TLR7 showed increased expression at transcriptional and proteome levels in XX and XXY, giving rise to significantly higher levels of IFNα/β expression, thus resulting in sex differences of autoimmune responses activation.^376,377^ The escape of TLR7 and TLR8 alleles from immune cells in X chromosome silences may make women more susceptible to autoimmune diseases.

CXorf21, as an X-linked type 1 IFN response gene, may escape X chromosome inactivation in immune cells.^378–380^ Compared with men, CXorf21 was more expressed in women. CXorf21 knockdown decreased TLR7-induced IFNA1mRNA expression and TNF-α and interleukin-6 secretion, thereby reducing autoimmune reaction.^381^ CXorf21 has been found in systemic lupus erythematosus and Sjögren’s syndrome susceptibility genes through GWAS studies, and its level has been discovered related to disease activity.^382–384^ In addition, elevated levels of the CXorf21 gene in female monocyte, B cell and lymphoblastic cell lines compared to male leaded to increased susceptibility to female autoimmune diseases.^385,386^ Therefore, further studies on CXorf21 and TLR7 genes in the future will make the potential mechanism of sex differences in autoimmune diseases more clear.

C-X-C motif chemokine receptor 3 (CXCR3), as an X-linked gene, can exhibit XCI escape. CXCR3 is also a chemokine receptor that is mainly expressed on activated CD4 and CD8 T cells and serves an adaptive immune function.^387^ Compared to XY T cells expressing CXCR3, XX T cells had increased levels of CXCR3 and activated phenotype.^388^ In addition, CXCR3 expression in CD4T cells of systemic lupus erythematosus patients was higher in women.^389^ CXCR3 enrichment was also found in active lupus nephritis patients CD4T cells, suggesting a correlation between CXCR3 and systemic lupus erythematosus.^390^ Additionally, CXCR3 was overexpressed in Sjögren’s syndrome patients salivary gland, and T cells infiltrate and accumulate in salivary gland, thereby accelerating Sjögren’s syndrome occurrence and development.^391,392^ Taken together, CXCR3 participated in the development of autoimmune diseases based on sex differences. For years, when scientists tried to clarify sexism in nature, hormones played a central role, but the results were either uncertain or even contradictory. To fill this knowledge gap, the scientific community has highlighted the recognition of these key genes and their effector protein products, thus promoting women’s susceptibility to autoimmune diseases.

#### Epigenetic modification

Some studies have found sex-specific transcriptome and methylation differences separated from X chromosome inactivation, implying that sexual dimorphism may influence autoimmune diseases progression via epigenetic mechanisms.

DNA methylation and demethylation participated in estrogen regulation of autoimmune diseases. In systemic lupus erythematosus and rheumatoid arthritis, increased DNA demethylation and ERα expression were observed in the proximal promoter region of ERα gene transcription site, suggesting that ERα overexpression may be associated with systemic lupus erythematosus and rheumatoid arthritis.^393^ In female systemic lupus erythematosus CD4+ T cells, estrogen down-regulated DNMT1 expression and enhanced the global DNA hypomethylation. ER antagonists saved DNMT1 and DNA hypomethylation down-regulated by estrogen, suggesting that estrogen seemed to play an important role in systemic lupus erythematosus development by regulating DNA hypomethylation.^394^ The decrease of DNA methylation at the X promoter site may lead to dose abnormality of X-linked genes in immune cells. The CD40LG promoter in CD4T cells from healthy men was unmethylated, whereas the CD40LG promoter in healthy women (XX) was partially methylated.^361^ As a result, 5’azacytidine-induced demethylation promoted higher CD40LG expression in healthy female CD4T cells than in male, resulting in an elevated autoimmune response. Systemic lupus erythematosus is an epigenetic disease marked by DNA methylation damage in T cells. CD4 T cells in systemic lupus erythematosus patients exhibited female-specific CD40LG promoter hypomethylation, leading to elevated CD40LG expression and inflammatory responses activation, thereby accelerating lupus activity progression.^361^ Taken together, demethylation of CD40LG may increase women’s susceptibility to lupus. Similarly, in female patients with systemic sclerosis, overexpression of CD40LG transcripts and proteins in CD4T cells was linked to reduced DNA methylation in the CD40LG promoter and enhancer regions.^363^

MicroRNAs (miRNA) made a critical impact on autoimmune diseases based on sex difference. MiRNAs, such as miR106A and miR-17-92, regulated inflammation-related genes such as interleukin-1 and TNF-α, leading to immune responses. It has been found that the X chromosome contains nearly 800 miRNAs, which was approximately ten times more than the Y chromosome.^395^ Therefore, sex differences in X-linked and immunomodulatory related miRNA expression can be used to explain why women have a stronger immune system.^396,397^ MiR-17-92 cluster located on the X chromosome has been shown to be a crucial element in the maturation of B and T cells, engaged in autoimmunity activation process.^398,399^ In addition, miR106a on the X chromosome has been shown to down-regulate interleukin-10, thereby inhibiting inflammatory response. In addition, E2 increased the activation of IFN-α signal transduction in B cells by decreasing let-7e-5p, miR-98-5p and miR-145a-5p expression and regulating kappa B kinase ε (IKKε) level in systemic lupus erythematosus.^400^ The expression of serine/arginine-rich splicing factor 1 (SRSF1) was decreased in systemic lupus erythematosus patients. Has-mir-10b-5p regulated SRSF1 transcription and down-regulated SRSF1 protein expression by inhibiting the 3’-UTR activity of SRSF1, resulting in T cell activity and enhanced autoimmunity.^401^ The expression of has-miR-10b-5p in healthy women T cells was higher than that of healthy men, and the expression of has-miR-10b-5p in systemic lupus erythematosus patients T cells was also higher, resulting in immune response activation.^401^ In conclusion, DNA methylation, demethylation, and miRNA regulation of immune response may help explain sex differences in autoimmune diseases. There are numerous differences between the immune responses of men and women. However, it is not until recent years that sex differences in the immune system have been properly recognized and studied. Considering the notable variations in immune response between the sexes, the importance of studying bisexual disease models is becoming more and more obvious.

### Clinical implications

The treatment of autoimmune diseases has different effects in men and women. There are sex-specific variations in how cancer patients respond to bevacizumab and rheumatoid arthritis patients respond to glucocorticoids, indicating that the efficacy of women is better than that of men.^402,403^

The main component of compound oral contraceptive (OCP) is 17α-ethinylestradiol, which is a synthetic analog of natural estrogen. However, considering the unpredictability and variability of systemic lupus erythematosus, it may not be safe for systemic lupus erythematosus patients to use OCP.^404,405^ Several studies revealed that patients exposed to OCP may develop systemic lupus erythematosus.^404,406–409^ However, two higher-quality randomized controlled trials (RCTs) found no correlation between OCP and systemic lupus erythematosus.^410,411^ A single-blind, non-placebo study found no significant difference in systemic lupus erythematosus disease activity, seizure incidence and time of first attack between groups receiving different types of contraceptive treatment, including OCP.^410^ The second double-blind randomized controlled trial in the safety (SELENA) study included 183 systemic lupus erythematosus patients who were randomized to receive OCP or a placebo for a year. Study indicated that the incidence of systemic lupus erythematosus did not differ between the two groups.^411^ As a result, the World Health Organization suggests that if antiphospholipid antibodies were not present or cardiovascular risk factors were unknown., most female systemic lupus erythematosus patients can use OCP.^404^ Most notably, though, OCP is disabled in female systemic lupus erythematosus patients who have a history of thrombosis or who have positive or unknown antiphospholipid antibody.^412,413^ Depending on the type of hormone used, dosage, and length of use, OCP’s impact and risk on systemic lupus erythematosus may change. Although it is theoretically feasible, OCP application should be fully discussed to balance each patient benefits and risks.

The best way to relieve menopausal symptoms is hormone replacement therapy.^414^ Large prospective cohort studies reported a causal relationship between hormone replacement therapy and an increased risk of systemic lupus erythematosus in postmenopausal women.^415^ Furthermore, Meier et al. found that a larger probability of developing systemic lupus erythematosus as the duration and cumulative dose of hormone use increased.^416,417^ According to several randomized controlled trials, hormone replacement therapy alone can not raise the risk of thrombosis or coronary heart disease in patients with inactive systemic lupus erythematosus who were antiphospholipid antibodies negative and had no history of thrombosis.^418^ However, most studies have found that hormone replacement therapy use in patients with active systemic lupus erythematosus may increase thrombotic events.^419,420^ In summary, hormone replacement therapy is not safe for systemic lupus erythematosus patients with antiphospholipid antibodies or previous vascular thrombosis events.^420^ Therefore, it is suggested that clinicians should formulate different treatment plans according to different sexes of patients, so as to better achieve the individualized treatment emphasized by precision medicine.^316^ Taken together, sex is a particularly factor to be considered in the autoimmune diseases randomized controlled trials in the future. A comprehensive sex stratification model, including large forward-looking data sets with genetics, biomarkers and treatments, will generate significant value in public health, prognosis and precision medicine.

## Sex difference in neurodegenerative diseases

### Introduction

Neurodegenerative diseases of the central nervous system are a class of neurological diseases that cause the central nervous system to gradually lose its neurons, impacting millions of people’s lives globally. It primarily encompasses Alzheimer’s disease, Parkinson’s disease, and amyotrophic lateral sclerosis, which have a significant impact and challenge for the public health system. Alzheimer’s disease is the most prevalent neurodegenerative disease. Alzheimer’s disease is distinguished by cognitive deterioration, memory loss, and loss of daily activity. Amyloid plaque and intracellular neurofibrillary tangle deposition are typical pathological and diagnosis features of Alzheimer’s disease. Furthermore, these pathological changes can cause neuronal apoptosis, cellular pathway damage, and a gradual decline in brain structure and function.^421–423^ The incidence of Alzheimer’s disease is expected to triple by 2050.^424–426^ It is worth noting that the incidence of Alzheimer’s disease in women is roughly double that of men, and that women’s progresses faster, whereas the biological causes of these differences are still unknown.^427^ The second most prevalent neurodegenerative disease is Parkinson’s disease, with an annual incidence of 0.014% in the general population and 0.16% among those aged 65 and older.^428^ Parkinson’s disease is primarily characterized by stiffness, tremors, impaired balance and coordination, slow movement, and potentially loss of movement.^429^ The hallmarks of Parkinson’s disease include the build-up of α-synuclein aggregates in intracellular pathways and the death of dopaminergic neurons in the substantia nigra.^430^ Parkinson’s disease could be caused by oxidative stress, mitochondrial dysfunction, and elevated reactive oxygen species production, which could lead to neuronal loss, α-synuclein misfolding, and aggregation.^431,432^ Importantly, the risk of developing Parkinson’s disease was 1.6–1.9 times higher in men than in women.^433^ Amyotrophic lateral sclerosis (ALS) is a rare disease whose incidence increases with age. The prevalence of ALS in men was higher than that in women, about 1.35 times higher.^434,435^ The primary characteristics of amyotrophic lateral sclerosis were progressive skeletal muscle weakness, muscle atrophy, muscle bundle fibrillation, medulla oblongata paralysis, and the pyramidal sign.

The aging brain is more susceptible to neurodegenerative diseases. Aging, as an important risk factor, affects men and women differently when it comes to NDs. Gur et al. discovered that the brain volume of the frontotemporal lobe was larger in males than in females using magnetic resonance imaging,^436^ whereas the distribution of brain atrophy in females was more symmetrical.^437^ Notably, postmenopausal women showed accelerated brain volume loss.^436^ Male and female brain metabolism seemed to begin at the age of 60 and was common in the frontal lobe, temporal lobe and anterior cingulate gyrus. However, there are location differences between the sexes in the brain metabolism. Men have slower brain metabolism in the frontal lobe, caudate nucleus, and cingulate gyrus, whereas women have slower metabolism in the occipital lobe, thalamus, and cerebellum. Furthermore, a decline in brain metabolism was observed in men after the age of 70, but not in women.^438^ Female-specific risk factors for Alzheimer’s disease include reproductive activity. In a population-based study, Jang et al discovered that women with more than five pregnancies were more likely to develop Alzheimer’s disease than those with fewer than five pregnancies or were not pregnant.^439^ Gilsanz et al. showed that earlier menopause increased dementia risk.^440^ These findings indicated that female reproductive behavior was strongly associated with the risk of developing Alzheimer’s disease.

### Mechanism

**Fig. 5:**
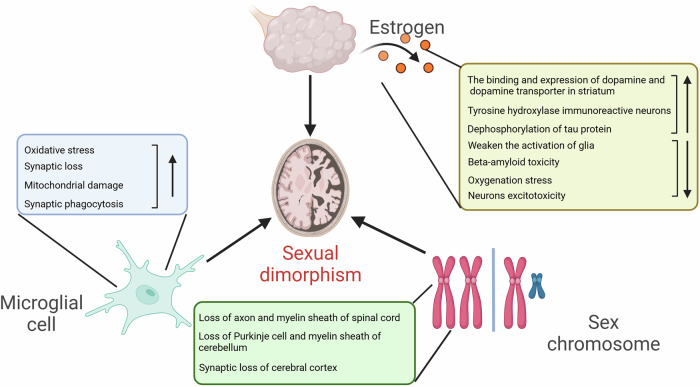
Mechanisms related to sex differences in neurodegenerative diseases: including estrogen, sex chromosomes and microglia. Compared with men, women have higher levels of estrogen. Estrogen can not only reduce the depletion of binding and expression of dopamine and dopamine transporter in the striatum, but also reduce the loss of tyrosine hydroxylase immunoreactive neurons and weaken glial activation. This effect makes estrogen play a protective role in Parkinson’s syndrome. In addition, estrogen also plays a protective role in Alzheimer’s disease. This is not only shown in that estrogen can not only induce dephosphorylation of tau protein and prevent hyperphosphorylation in its neurons, but also protect neurons from β-amyloid toxicity, oxidative stress and excitotoxicity. In addition, activation of gray matter microglia plays a key role in neurodegenerative diseases, which can not only lead to synaptic phagocytosis and synaptic loss, but also lead to oxidative stress and mitochondrial damage. The accumulation of brain-specific genes on the X chromosome in the sex chromosome puts them in a unique position that affects the response of the central nervous system to injury. X chromosome can cause the loss of axon and myelin sheath of spinal cord, Purkinje cell and myelin sheath of cerebellum and synapse loss of cerebral cortex. This figure was created with the aid of BioRender (https://biorender.com/)

Sex differences in neurodegenerative diseases result in different risks and pathological manifestations, whereas the underlying mechanisms are not fully clarified. Sex hormones, sex chromosomes, and microglia participate in the regulation of neurodegenerative disease progression (Fig. 5).

#### Hormone regulation

Recent animal and human studies have provided additional evidence that Alzheimer’s disease may be prevented by estrogen. Yue et al. found that estrogen-deficient APP23 transgenic mice had significantly lower brain estrogen levels and increased β-amyloid peptide deposition compare to APP23 transgenic mice. Study has indicated that brain estrogen depletion may be a significant risk factor for the neuropathology of Alzheimer’s disease.^441^ It has been shown that estrogen can dephosphorylate tau protein and stop its neurons from becoming hyperphosphorylated, which delayed the onset of the brain degenerative changes linked to Alzheimer’s disease.^442^ In addition, estrogen enhanced Akt activation and induced the death signals GSK-3β and BAD phosphorylation and inactivation in neurons, thereby protecting neurons survival and reducing neurodegenerative diseases occurrence.^443–445^ Many studies revealed that estrogen had a protective effect against Alzheimer’s disease by protecting neurons from amyloid beta toxicity,^444,446,447^ inhibiting oxidative stress,^448–450^ and alleviating neuronal excitatory toxicity induced by n-methyl-D-aspartate.^451,452^ Aromatase gene is an important gene for the synthesis of estrogen in vivo.^453^ Ishunina et al showed that the expression of ERα and aromatase in the hippocampus of women increased with age, while the expression of these genes decreased in women with Alzheimer’s disease, leading to reduced brain estrogen production and increased risk of Alzheimer’s disease.^454^ To summarize, estrogen plays a protective effect on the treatment of Alzheimer’s disease, which warrants further investigation in the future.

Ragonese et al. confirmed that Parkinson’s disease was related to the decrease of estrogen stimulation.^455^ Currie et al.^456^ also found that postmenopausal women who took estrogen supplements had a decreased likelihood of Parkinson’s disease compared to those who did not.^456^ In addition, it has recently been shown that the single nucleotide polymorphism allele of the estrogen β receptor was more prevalent in patients with earlier occurrence of Parkinson’s disease.^457^ Estrogen can prevent MPTP-induced depletion of dopamine expression and binding in the striatum, reduce the loss of tyrosine hydroxylase immunoreactive neurons, and weaken MPTP-induced glial activation.^458–460^ D’Astous et al found that ERα agonist (PPT) reduced striatal dopamine depletion induced by MPTP, suggesting that ERα contributed to the neuroprotective effect of estrogen induced by MPTP.^461^ Furthermore, it has been demonstrated that estrogen decreased MPTP-induced dyskinesia in monkeys.^462^ Quesada et al. discovered that estrogen interacted with IGF-1 to protect dopaminergic neurons in the substantia nigra and striatum and maintain the motor function of animals with 6-hydroxydopaminergic lesions.^463^ Short-term estrogen therapy increased the availability of dopamine transporters in postmenopausal women’s caudate putamen.^464^ In addition, estrogen inhibited the synthesis of free radicals and protected striatal neurons from oxidative stress, thereby reducing the occurrence of Parkinson’s disease.^449,450^ Therefore, both animal and human studies have indicated that estrogen provided a positive regulatory effect on the dopaminergic system in the substantia nigra and striatum, which may be the basis of estrogen’s protective role in Parkinson’s disease. Collectively, these data are shown in that estrogen performed a variety of regulatory functions in synaptic plasticity, neuronal growth, memory formation and neuroprotection. Although the precise mechanism of estrogen’s neuroprotective effect was unclear, it appeared to mediate the upregulation of growth factor production, synapses formation, and the activation of anti-inflammatory and antioxidant pathways. These results implied that neurodegenerative diseases treatment required an understanding of the interplay between genetic and hormonal regulation.

#### Sex chromosome regulation

Compared to different somatic tissues, the proportion of genes on the X chromosome was significantly higher than that on the autosomal chromosome, and they were expressed preferentially in the brain.^465^ In the evolution process, brain-specific genes accumulation on the X chromosome placed them in a special position that influenced how the central nervous system reacted to injury. In multiple sclerosis, activation, demyelination, axonal damage, and synaptic loss of microglia and astrocytes were the central nervous system’s responses to immune attacks.^466^ Trapp et al.^467^ showed experimental allergic encephalomyelitis (EAE) was an autoimmune disease mainly involved in specifically sensitized CD4 + T cells and characterized by mononuclear cell infiltration and demyelination around small blood vessels in the central nervous system. It is an ideal mice model for human multiple sclerosis. The FCG model was used to show the sex chromosome complement effect in the central nervous system during EAE.^468^ It was found that compared with XX, EAE mice with XY-sex chromosome complement in central nervous system showed more severe EAE, with pathological manifestations of axon and myelin loss in the spinal cord, purkinje cells and myelin loss in the cerebellum, and synaptic loss in the cerebral cortex. This is the first evidence of sex chromosomes’ influence on neurodegenerative diseases. Human clinical observations also showed that Multiple sclerosis was more common in women (XX), whereas men (XY) had more serious disease progression.^469,470^ Aβ deposition is an essential component of pathological changes in Alzheimer’s disease. APP influenced the Aβ synthesis because it was the upstream precursor protein of Aβ. SP can result from Aβ protein overexpression., which affected the development of Alzheimer’s disease pathological process. In the Alzheimer’s disease model of mice expressing human APP (hAPP), the FCG model showed that the mortality and cognitive impairment of mice with XY- sex chromosome complement were more serious than those of XX.^471^ In humans, the genetic variant of KDM6A was expressed elevated in the brain and was linked to enhanced cognitive function in aging and preclinical Alzheimer’s disease, which in turn improved human brain health. KDM6A is a histone demethylase gene that escapes X chromosome inactivation. Davis et al found that KDM6A knockdown in XX neurons aggravated amyloid beta-mediated neuronal toxicity. Therefore, the study indicated that KDM6A had a significant effect in the sex chromosome, helping to reduce mortality and prevent brain dysfunction in Alzheimer’s disease.^471^

#### Microglia cell and gene regulation

Microglia were activated in both white and gray matter and played a vital role in neurodegenerative diseases.^472–474^ Alzheimer’s disease and brain aging pathophysiological process were involved with microglia activation.^475,476^ Among multiple sclerosis patients, men had more pathological magnetic resonance imaging markers than women in white matter lesions on magnetic resonance imaging, including a higher proportion of T1 “black holes” and increased longitudinal differences in measurements of demyelination and axonal integrity on diffusion tensor imaging.^477^ Microglia, the central nervous system’s resident immune cells, can have both beneficial and harmful effects during normal and abnormal pathological status.^478,479^ Microglia were believed to be advantageous in the early stage of Alzheimer’s disease and harmful in the later stage.^480,481^ Activated gray matter microglia can cause synaptic phagocytosis and loss.^474,482^ Apolipoprotein E genotype and sex are two recognized Alzheimer’s disease risk factors for regulating microglial function. The study found that women with APOE4 carriers had increased affinity for pro-atherosclerotic associated lipoproteins, increased ROS production and tau accumulation, and decreased ability to clear Aβ, leading to the predisposition of APOE4 carriers to cardiovascular diseases and neurodegenerative diseases.^483^ By comparing the interaction between microglia and amyloid plaques in EFAD mice with APOE3 and APOE4 genotypes at 6 months, it was found that the microglia coverage of plaques was the highest in male APOE3 mice, while the highest amyloid level and low microglia coverage were shown in APOE4 genotypes and women. This suggested that APOE4 genotypes and women had an elevated risk of Alzheimer’s disease.^481,484^ Furthermore, higher levels of Aβ aggregates may activate microglia, induce oxidative stress, mitochondrial damage, and synaptic loss, increasing the possibility of developing Alzheimer’s disease.^484,485^

As microglia were related to the numerous age-related neurodegenerative diseases pathogenesis of different sexes, everyone pays more and more attention to the regulation of sex differences in microglia throughout the life cycle. However, more researches were needed to determine the regulatory mechanisms that microglia-mediated sex effects throughout the life cycle. In short, the prevention and management of age-related brain diseases based on microglial responsiveness will become a promising treatment approach. In the future, microglia will be proved to be a potential target for regulating sex-specific inflammatory signal transduction. The identification of upstream regulatory mechanisms (hormones, chromosomes or their interactions) will guide the development of sex-specific treatment.

### Clinical implications

With the aggravation of the aging of the population, neurodegenerative diseases have an increasing influence on human health. At the moment, the fact that sex disparities existed in the neurodegenerative diseases clinical management cannot be disregarded. Therefore, sex-specific treatment is critical for improving the prognosis of these diseases. Several disease improvement drugs for Alzheimer’s disease are being studied worldwide, but only a few studies have conducted sex stratification analysis.^486^ Numerous scientific investigations have demonstrated that estrogen treatment can postpone the onset of Alzheimer’s disease.^487–493^ The risk of Alzheimer’s disease was estimated to be reduced by 29–44% as a result of estrogen therapy.^490,491^ Therefore, estrogen can be expected to become an important treatment for Alzheimer’s disease, especially for estrogen deficiency in menopausal women or men may be better treatment. The study found that the use of nivaldipine alleviated the progression in patients with mild Alzheimer’s disease when compared to placebo, however, the study did not include a statistical analysis of sex.^494^ Furthermore, women who took high-dose statins had a decreased risk of developing Alzheimer’s disease., but not in black men. To summarize, statin use in Alzheimer’s disease is influenced by sex and race/ethnicity.^495^ In a META study, Avgerinos et al. found that application of antibodies against Aβ in Alzheimer’s disease did not show differences in sex and apoE genotype.^496^ The Multidomain Alzheimer’s Prevention Trial and the Finnish Geriatric Intervention Study for the Prevention of Cognitive Impairment and Disability did not assess variations in outcomes based on sex.^497,498^ Therefore, the study of sex differences related to the treatment of Alzheimer’s disease patients remains to be explored more widely.

Currently, there are no sex-specific treatment recommendations for Parkinson’s patients.^499^ However, levodopa pharmacokinetics have been observed to differ by sex in some studies, with females exhibiting a markedly greater area under the curve and maximum plasma concentration in comparison to males. Only women can significantly predict curve and maximum plasma concentration, which may be connected with the higher bioavailability of levodopa in women.^500,501^ Sex differences in pharmacokinetics and pharmacodynamics often resulted in more frequent levodopa-induced dyskinesia in women, so women should consider low-dose levodopa therapy.^502^ In addition, men may receive higher doses of levodopa for Parkinson’s disease because of genetic mutations in coding enzymes and their involvement in dopamine metabolism.^503^ Deep brain stimulation (DBS) in the globus pallidus nucleus or subthalamic nucleus is more effective in treating late-stage Parkinson’s disease with dyskinesia or drug intolerance.^504^ After DBS treatment, both men and women had the same improvement in the score of UPDRS-III withdrawal. Women improved more than men in daily life activities, and had a favorable impact on the comprehensive scores of motor ability, cognition and PDQ39, which were not shown in men.^505^ Similarly, Accolla et al estimated that one month before DBS and 11-14 months after DBS, the response of women with motor retardation to DBS was lower than that of men, while women improved more in daily living activities after DBS.^506,507^ Therefore, DBS treatment may help to enhance Parkinson’s disease patients clinical prognosis, especially for female Parkinson’s disease patients. However, there is no clear evidence of sex differences in the efficacy or side effects of anti-Parkinson’s drugs, including levodopa. Data regarding sex variations in exercise and non-exercise outcomes following DBS were also lacked. Further study will be needed to explore how sex differences affect the drug response to Parkinson’s disease. Therefore, it is necessary to broaden our comprehension of sex differences and to identify individuals at risk of Parkinson’s disease early so as to ensure individualized treatment.

Amyotrophic lateral sclerosis is an incurable disease, with current treatments consisting of two disease palliative medications (riluzole and Edaravone) and supportive multidisciplinary therapy.^508,509^ In a clinical trial of sex hormone drugs, tamoxifen was found to be associated with a lower risk of ALS in women, while testosterone was associated with a higher risk.^510^. Furthermore, Nefussy et al. discovered that statins caused faster progression in amyotrophic lateral sclerosis than in amyotrophic lateral sclerosis patients who did not take statins. Statins accelerated female amyotrophic lateral sclerosis progression compare to males.^511,512^ As a result, statins may be ineffective for treating amyotrophic lateral sclerosis patients.

In a word, basic and clinical research that incorporates sex differences into disease mechanisms can contribute to better identify causes and provide new therapies. Many current study results do not show sex differences in neurodegenerative diseases treatment regimens, resulting in limited meta-analyses. Women were underrepresented in basic and clinical trials, so many findings can not fully reflect the effect of sex on neurodegenerative disease pathogenesis and treatment. In addition, a variety of societal, economic, and biological factors also impeded research into sex disparities. In the future, these issues will be needed to receive more attention and solutions, and we hope to find out effective sex-specific treatment strategies in as many studies as possible.

## Conclusion and perspective

In summary, angiotensin receptor-neprilysin inhibitors, aldosterone receptor antagonists, sodium-glucose co-transporter 2 inhibitors, ivabradine, and soluble guanylate cyclase stimulators have not shown significant differences in treating cardiovascular dysfunction between men and women. Further large-scale clinical trials are needed to explore these aspects. However, the clinical applications of estrogen and androgen therapy, as well as novel oral antidiabetic drugs (GLP-1 receptor agonists, SGLT2 inhibitors), are crucial for regulating different sexes glucose homeostasis and improving metabolic abnormalities. Hormone replacement therapy and immunotherapy may also play distinct roles in improving the prognosis of tumors such as liver cancer, lung cancer, and prostate cancer due to sex differences. PLD combined with carboplatin and selective inhibitors targeting PDE 10 A and SIK2 may offer promising treatment strategies for refractory recurrent ovarian cancer and other cancers. In the future, more drugs are expected to undergo extensive basic and clinical trials in different sexes to better identify effective treatment options, which are crucial for maintaining metabolic balance and inhibiting tumor progression. Among the sex differences observed in autoimmune diseases, women predominate in systemic lupus erythematosus and Sjogren’s syndrome. The roles of estrogen, X chromosome and epigenetic regulation, to a certain extent, contributes to women’s susceptibility to autoimmune diseases, which provides new directions for clinical treatment strategies. At present, the application of OCP in the active phase of female autoimmune diseases still needs to be cautious, not only systemic lupus erythematosus. Therefore, more valuable clinical studies deserve our implementation, and the specific mechanism of sex differences in autoimmune diseases also deserves our further attention and research. Additionally, increasing evidences suggest differences in the incidence and progression of neurodegenerative diseases between women and men. Here, we summarize the sex differences embodied in the mechanisms and treatments associated with estrogen, microglia, and sex chromosomes that cause neurodegenerative diseases. The impact of neurodegenerative diseases on the aging population is increasing. Undoubtedly, sex differences are of great significance to clinical research and practice. However, research on sex differences in neurodegenerative diseases is still relatively scarce, especially among women. In the future, sex should be considered as an important variable to study in the progression of neurodegenerative diseases.

In conclusion, there are unique characteristics in the epidemiology, etiology, pathophysiological manifestation, and treatment of patients with different diseases related to sex. Sex differences in human diseases are influenced by an individual’s unique interaction of sex hormone status, sex chromosomes, and environment. Therefore, we should pay more attention to these factors and develop comprehensive management plans and prevention strategies based on their specificity to reduce the adverse effects of related risk factors on sex related diseases patients, thereby improving clinical diseases symptoms and prognosis. In the future, we hope to conduct larger-scale basic and clinical studies on sex differences in cardiovascular, metabolic, cancer, autoimmune, and neurodegenerative diseases. More representative women need to be included in clinical studies (phase I, II, and III) to observe the effects and differences in the efficacy and safety of drug therapy across sexes through analysis of outcomes between sexes and diseases. This contributes to further exploring and revealing various systemic diseases molecular mechanisms, as well as developing more precise individualized strategies for the prevention and treatment of sex-related diseases, which will better guide clinical treatment.
